# Persistent Low-Grade Inflammation and Post-COVID Condition: Evidence from the ORCHESTRA Cohort

**DOI:** 10.3390/biomedicines14010083

**Published:** 2025-12-31

**Authors:** Elisa Gentilotti, Carolina Alvarez Garavito, Anna Górska, Roy Gusinow, Lorenzo Maria Canziani, Pasquale De Nardo, Alessandro Visentin, Maria Giulia Caponcello, Michela Di Chiara, Aline-Marie Florence, Gerolf de Boer, Salvatore Cataudella, the ORCHESTRA Study Group, Gabriel Levy Hara, Adriana Tami, Maddalena Giannella, Cédric Laouénan, Jan Hasenauer, Jesús Rodríguez-Baño, Evelina Tacconelli

**Affiliations:** 1 Infectious Diseases, Department of Diagnostics and Public Health, University of Verona, 37129 Verona, Italy; 2 Life and Medical Sciences (LIMES), University of Bonn, 53113 Bonn, Germany; 3 Bonn Center for Mathematical Life Sciences, University of Bonn, 53113 Bonn, Germany; 4 Infectious Diseases and Microbiology Unit, Hospital Universitario Virgen Macarena and Department of Medicine, University of Sevilla/Biomedicines Institute of Sevilla, CSIC, 41009 Sevilla, Spain; 5 CIBERINFEC, Instituto de Salud Carlos III, 28029 Madrid, Spain; 6 Infectious Diseases Unit, Department for Integrated Infectious Risk Management, IRCCS Azienda Ospedaliero-Universitaria di Bologna, 40138 Bologna, Italy; 7 Université Paris Cité, INSERM IAME UMR 1137, 75018 Paris, France; 8 AP-HP Nord, Hôpital Bichat, Department of Epidemiology Biostatistics and Clinical Research, 75018 Paris, France; 9 University Medical Center Groningen, Department of Medical Microbiology, and Infection Prevention, University of Groningen, 9713 GZ Groningen, The Netherlands; 10 CINECA Interuniversity Consortium, 40126 Bologna, Italy; 11 Instituto Alberto Tachini, School of Medicine, University of Buenos Aires, Buenos Aires C1123AAR, Argentina; 12 Department of Medical and Surgical Sciences, Alma Mater Studiorum University of Bologna, 40138 Bologna, Italy

**Keywords:** Post-COVID-19 condition, laboratory diagnostic, biochemistry, persistent low-grade inflammation

## Abstract

**Background:** Persistent low-grade inflammation has been proposed as part of the biological mechanisms underlying post-COVID condition (PCC), which can result in laboratory tests abnormalities. However, the accuracy of routine laboratory tests for the diagnosis and follow-up of PCC is still under discussion. **Methods:** Patients with SARS-CoV-2 infection enrolled in the prospective, multinational ORCHESTRA cohort study, which included both European and non-European countries, were followed up for 18 months after acute infection. Blood test results were collected at acute infection and at 6, 12, and 18 months. A multivariable analysis was performed to estimate the relationship between the alterations of biochemical markers and the presence of four distinct PCC phenotypes, identified previously through a principal component analysis—respiratory (RESc), chronic pain (CPc), chronic fatigue (CFc), and neurosensorial (NSc)—during follow-up. Furthermore, this study investigated the correlation between biochemical parameters measured during the acute phase and the subsequent development of PCC. Finally, the relationship between the severity of the acute infection and biochemical abnormalities observed during follow-up was assessed. **Results:** The cohort included 4587 patients, 58% male, with a mean age of 58.7 (±15.5) years. A robust multivariable analysis demonstrated that, compared to controls, patients with PCC, and in particular those in the RESc cluster, presented higher mean C-reactive protein (CRP) levels at the 12- and 18-month follow-up (*p*-value = 0.01). In each follow-up, CRP values in patients with PCC and RESc were above 3 mg/L, corresponding to those observed in low-grade inflammation (3–10 mg/L). The severity of COVID-19 acute infection was associated with increased levels of CRP, ferritin and LDH during follow-up (*p* < 0.001). Biochemistry abnormalities detected during the early stages of acute COVID-19 did not correlate with an increased risk of developing PCC and its phenotypes. **Conclusions:** In patients with the RESc PCC phenotype, identified through a principal component analysis, blood test abnormalities consistent with prolonged and sustained low-grade inflammation can be detected up to 18 months after acute infection, supporting its role in the pathogenesis of PCC. Based on these results, trials on anti-inflammatory drugs, together with symptom-tailored interventions for patients with RESc, should be planned to prove their effectiveness in managing PCC and improving patient outcomes.

## 1. Background

Post-COVID-19 condition (PCC), also referred as “post-COVID-19 syndrome” [1], “post-acute sequelae of COVID-19” [2], or “long COVID-19” [3,4], imposes a significant physical and psychological burden, profoundly impacting patients’ well-being across multiple domains, including health, social behaviour, quality of life, and productivity [5]. According to the WHO’s definition, PCC occurs in individuals with a history of probable or confirmed SARS-CoV-2 infection, usually within three months from diagnosis, with symptoms that last for at least two months and cannot be explained by an alternative diagnosis [3]. Currently, diagnostic tools are failing to detect many clinical manifestations of PCC, limiting both decision making and clinicians’ confidence in the available epidemiological estimates [6]. The absence of validated tools to diagnose and assess PCC also hampers the understanding of underlying biological mechanisms and complicates efforts to develop effective management and treatment strategies. Several diagnostic tools proved to be promising for some of the clinical manifestations of PCC, such as the use of psychophysical tests for the diagnosis and characterisation of chemosensory dysfunction [7], sublingual videomicroscopy to demonstrate PCC-related multisystemic vascular disease [8], and lung MRI with hyperpolarised Xenon gas for monitoring longitudinal change in lung function [9]. Bio-humoral parameters assessed during the early stages of the viral infection [10] have been associated with a higher risk of disease progression toward more serious clinical conditions and short-term unfavourable outcomes, such as acute respiratory distress syndrome, disseminated intravascular coagulation, multiple organ failure [11], and intensive care unit (ICU) admission [12,13,14,15]. In the context of PCC, investigators have focused on the assessment of pro-inflammatory biomarkers, such as cytokines and chemokines, to identify chronic dysregulation of immune/inflammatory responses to the acute infection as potential biological mechanisms underlying symptom persistence [16]. Although essential to clarify pathophysiological mechanisms, these laboratory tests are difficult to introduce into clinical practice [17,18,19]. The role of routine laboratory tests has also been discussed, suggesting that an increase in inflammatory biomarkers in the context of low-grade chronic inflammation may be a measurable signal of PCC [20]. However, the strength of conclusions is limited by the short-to-medium follow-up, the substantial heterogeneity of the study design and methodology, and model robustness.

The ORCHESTRA long-term sequelae study enrolled a multinational cohort of patients diagnosed with SARS-CoV-2 who were prospectively followed up with multiple assessments, including laboratory analysis. The present study reports the results of a wide range of biochemistry parameters collected within the ORCHESTRA long-term sequelae study, including liver and kidney function, haematological features, acute-phase proteins, and coagulation patterns. Laboratory tests were performed during the acute infection and at subsequent time points. A robust statistical analysis was performed to estimate the relationship between alterations in biochemical markers and the presence of four distinct PCC phenotypes, respiratory (RESc), chronic pain (CPc), chronic fatigue (CFc), and neurosensorial (NSc), during a 6-to-18-month follow-up period. These phenotypes were previously identified using an unsupervised machine learning algorithm based on principal component analysis (PCA) [21]. Furthermore, this study investigated the relationship between biochemical parameters measured during the acute phase and the subsequent development of PCC.

## 2. Methods

### 2.1. Study Design, Participants, and Procedures

This prospective cohort study enrolled patients with SARS-CoV-2 infection from February 2020 to March 2023 in five European and non-European countries participating in the ORCHESTRA COVID-19 long-term sequelae cohort: France, Italy, Netherlands, Spain, and Argentina. Inpatients and outpatients aged > 14 years with a laboratory-confirmed SARS-CoV-2 infection were enrolled after written informed consent and followed up at 6, 12, and 18 months after acute infection. During each follow-up visit, a clinical assessment was performed by qualified medical staff, and biological samples were collected for laboratory testing. The clinical assessment consisted of an anamnestic interview evaluating demographic and epidemiological features, pre-existing clinical conditions, ongoing chronic treatment, vaccination status, and acute infection characteristics, including hospitalisation, ICU transfer, oxygen therapy, early treatment for SARS-CoV-2, steroid therapy, and any other treatment administered. The quality of life (QoL) was assessed at each time point through the 36-Item Short Form Survey (SF-36) questionnaire [22,23]. Biological sampling was performed and samples were processed at each cohort’s local laboratory to assess the following biochemical parameters: haemoglobin, white blood cell count (WBC), lymphocyte count, neutrophil count, platelets, sodium, potassium, creatinine, glucose, haemoglobin A1c, total bilirubin, alanine aminotransferase (ALT), aspartate aminotransferase (AST), gamma glutamyl transpeptidase (GTT), albumin, lactate dehydrogenase (LDH), ferritin, creatine kinase (CK), fibrinogen, INR, partial thromboplastin time, D-dimer, NT-pro-BNP, troponin, C-reactive protein (CRP), procalcitonin (PCT), venous lactate, and complete urine tests. The protocol of this study is available on the ORCHESTRA website [24]. This study was approved by each cohort’s local ethics committee and by the centre (3199CESC). A detailed description of the study methodology is available in the Supplement (Sections S1 and S2). This study is registered on ClinicalTrial.gov (ID: NCT05097677) and the protocol is available at the institution website.

### 2.2. Data Collection

Study data were collected and managed using the REDCap electronic data capture tool (Research Electronic Data CAPpture). Since the follow-up of the cohorts enrolled in France and the Netherlands started before the ORCHESTRA project was financed (in February and March 2020, respectively), data from these two cohorts went through a post-data collection harmonisation process under the supervision of the Charité, Universitätsmedizin Berlin, and transformation by the Centre Informatique National de l’Enseignement Superieur [25,26].

According to the WHO definition, PCC was diagnosed in cases of continuation or development of new symptoms three months after the initial SARS-CoV-2 infection, with these symptoms lasting for at least two months with no other explanation [3]. Additionally, in this study, PCC was also diagnosed based on clinician evaluation in cases of significant worsening of pre-existing symptoms after the acute infection, in accordance with recent literature [4]. PCC severity was defined based on the impact of each combination of clusters on the QoL, which was assessed through the physical and mental component scores of the SF-36 questionnaire [22,23]. The questionnaires were scored using the PRO CoRE software developed by QualityMetrics, which applies US1998 norms. As stated above, PCA was used to identify clusters of symptoms, while logistic regression was used to perform an analysis of factors associated with PCC and each of the clusters: respiratory (RESc), neurosensorial (NSc), chronic fatigue-like (CFc), and chronic pain (CPc). A detailed description of the methods is available in a previous publication [21] and reported in the Supplement (Section S1).

### 2.3. Statistical Analysis

A full description of the statistical methodology is available in the Supplement. In the following paragraphs, the statistical analysis is summarised.

### 2.4. Univariable Statistical Analysis

An univariable analysis was conducted by computing Fisher’s exact test against the four PCC clusters at any time between 6 and 18 months for categorical covariates. Crude odds ratios (ORs) with 95% confidence intervals (95% CIs) and *p*-values were computed [27]. Differences in biochemical values were investigated at each time point between patients with and without PCC clusters during follow-up. All biochemical values were tested for normality using the Shapiro–Wilk test and, as some of them were not normally distributed, comparisons were performed with the Kruskal–Wallis test.

### 2.5. Filtering, Normalisation, and Transformation

Prior to fitting the multivariable models, we applied a systematic approach to select, filter, and prepare covariates, addressing the challenges posed by missing data and variable distributions. Briefly, we first selected all covariates with statistically significant univariable associations, then we excluded those with over 60% missing data and proceeded with complete-case analysis (Supplement, Figure S5) [28]. Imputation was not performed. Biochemical markers were subsequently normalised against their clinical reference ranges.

### 2.6. Acute-to-Follow-Up Predictive Models

To assess the predictive value of biochemical measurements during acute infection on the development of a specific PCC phenotype at follow-up time points (6, 12, and 18 months), and to reduce the size of the covariate space, we employed a logistic regression model with L1 regularisation (LASSO). Five distinct outcome variables were defined, each representing the presence or absence of a specific PCC phenotype at 6, 12, or 18 months after acute infection, along with a general PCC outcome. Covariates were systematically selected based on univariate analysis outcomes and the predefined filtering threshold for missing data. The selected covariates encompassed demographic factors, underlying medical conditions, acute-phase symptoms, hospitalisation status, cohort membership, COVID-19 treatment interventions, and a refined set of biochemical markers. A regularised logistic regression model was fitted across a sequence of regularisation parameters (λ). For each λ, the Akaike Information Criterion (AIC) was calculated using the model’s log-likelihood and the number of non-zero coefficients. The λ that minimised the AIC was selected. Covariates with non-zero coefficients at the optimal λ were retained and used to construct a reduced logistic regression model without penalisation, yielding final estimates and inference.

### 2.7. Longitudinal Modelling of Biochemical Markers and PCC Symptoms

To examine longitudinal associations between biochemical markers and PCC-related symptoms at 6-, 12-, and 18-month follow-up intervals, we implemented a penalised Generalised Linear Mixed-Effects Model (GLMM) following the method described by Groll and Tutz (2014), incorporating an L1 penalty (LASSO) within the log-likelihood to promote sparsity in fixed-effect estimates [29,30,31]. Consistent with the acute-to-follow-up analysis, we created five distinct binary response vectors representing the presence or absence of general PCC or specific PCC symptom clusters at each follow-up interval. Covariates included demographic factors, underlying medical conditions, acute-phase symptoms, hospitalisation status, cohort membership, COVID-19 treatment interventions, and selected biochemical markers measured concurrently at 6-, 12-, and 18-month follow-up visits. These covariates entered the models as fixed effects after applying the same filtering strategy as described previously. To account for repeated measurements within individuals, patient-specific random intercepts were included as random effects, effectively modelling within-subject correlations across time points.

### 2.8. Or for Changes in Biochemical Markers

To help with the interpretation of the relationship between biochemical markers identified as significant in our models and PCC, we performed a straightforward odds-ratio analysis based on changes in biochemistry levels. For each significant biomarker (e.g., CRP, lymphocytes), we defined a clinically relevant range of values commonly observed. We then analysed every possible change from one biochemical value to another. Each change was represented by computing the numerical difference between the two biochemistry values after normalisation and log10-transformation, as previously described. Using these computed differences and the corresponding regression coefficients from our mixed effects models, we derived the odds ratios for each change in a biochemistry level. These odds ratios indicate the odds of developing PCC or a particular phenotype when the biochemical variable increases or decreases from one clinical value to another. 

Finally, we visualised these odds ratios in heatmaps, providing a clear depiction of the increase or decrease in risk associated with changes in each biomarker level.

The data wrangling, univariable statistical tests, and visualisations were conducted in Python v.3.11.4, using the scipy v. 1.10.01 [18] and matplotlib v. 3.7.2 packages. The GLMM models were conducted in R v. 4.3.2 using lme4 v. 1.1-35.1, whereas the regularised GLMM models were conducted using glmmLasso v. 1.6.3 [29]. Regularised logistic regression models were implemented using glmnet v. 4.1-8 [32].

## 3. Results

### 3.1. Cohort Description

**Figure 1:**
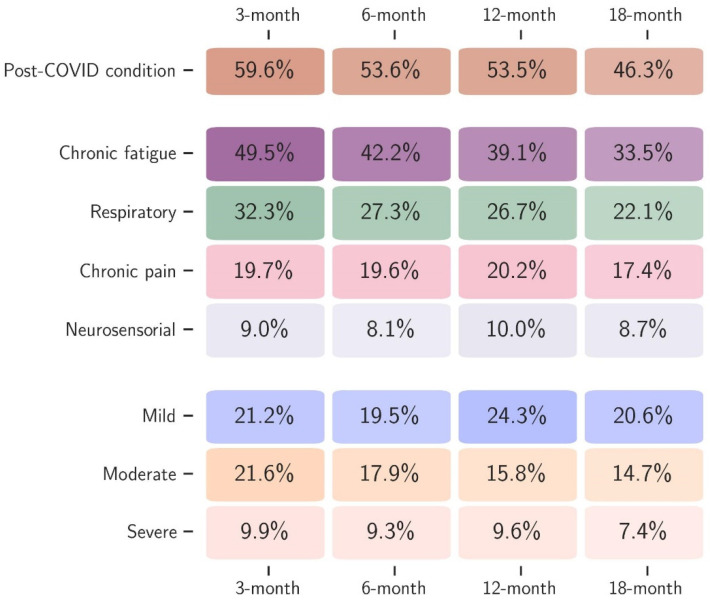
Proportion of patients diagnosed with PCC and the four clusters (chronic fatigue, respiratory, chronic pain, and neurosensorial), together with the severity of PCC according to the quality of life (mild, moderate, and severe) at each follow-up time point (3, 6, 12, and 18 months after acute infection).

This study included 4587 patients enrolled between February 2020 and March 2023. The mean age was 58.7 (±15.5), with slightly more male patients (2661, 58%). As many as 798 (18%) patients were vaccinated before the acute infection. Hospitalisation occurred in 3521 (77%) patients, with 1030 (22%) requiring ICU admission. A diagnosis of PCC was made in 1706 (60%), 1715 (54%), 1243 (54%), and 855 (47%) patients at the 3-, 6-, 12-, and 18-month post-acute infection visit, respectively. Overall, the most reported symptom during acute infection was asthenia (2781, 64%), followed by dyspnoea (2475, 56%), myalgia (1463, 35%), and arthralgia (763, 18%). Based on an unsupervised machine learning algorithm, four distinct clusters were identified during follow-up, with CFc being the most frequently reported at each time point, followed by RESc, CPc, and NSc (Figure 1). Table 1 summarises demographic, clinical, and epidemiological characteristics for the subsamples used to fit each outcome-specific model. Sample sizes reflect the number of patients within that cluster for whom all variables are available.

### 3.2. Univariable Analysis of Demographic, Clinical and Biochemical Factors Associated with PCC Between 6 and 18 Months After Acute Infection

We conducted an univariable analysis to assess the relation between demographic, clinical, and epidemiological features and the occurrence of PCC phenotypes to inform multivariable analysis.

As per the laboratory parameters, univariable models showed that the CRP values were higher during acute infection in patients who subsequently developed PCC (86.7 mg/L vs. 58.3 mg/L, *p* < 0.001). A prolonged increase of CRP values was also observed in patients with PCC at 12- and 18-months after acute infection compared to patients without PCC (3.7 mg/L vs. 2.4 mg/L, *p* < 0.001; 3.7 mg/L vs. 2.7 mg/L, *p* < 0.001). A cluster-specific analysis revealed a similar trend for CRP in patients with RESc, CPc, and CFc, but not in patients with NSc. Other statistically significant associations (*p* < 0.05) between biochemical features and PCC phenotypes are reported in the Supplement (Section S3, Tables S4–S9).

### 3.3. Multivariable Analysis of Demographic, Clinical and Biochemical Factors Associated with PCC Between 6 and 18 Months After Acute Infection

A descriptive analysis of the covariates included in each model is available in the Supplement (Supplement, Section S3, Tables S10 and S11).

#### 3.3.1. Analysis of the Impact of Biochemical Features Measured During the Acute Infection on PCC Development

The results from models incorporating all acute-phase variables revealed no evidence of an association between biochemical parameters measured during the acute infection and the presence of PCC at any time point between 6 and 18 months post-infection. Notably, in the context of regularised logistic regression, the exclusion of acute-phase biochemical variables from the final models suggests that their coefficients were shrunk to zero in the penalised model that minimised the AIC. This indicates that their inclusion in the model did not contribute meaningfully to the prediction of PCC or its symptom clusters (Supplement, Section S3, Table S12).

#### 3.3.2. Analysis of the Impact of Biochemical Features Measured During Follow-Up on PCC Development 

The results of the multivariable analyses incorporating longitudinal (time-point-to-time-point) PCC data are shown in Table 2. Compared to controls, the patients with PCC in our sample presented an independent higher mean lymphocyte count (OR: 3.12; 95% CI: 1.05–9.28; *p*-value = 0.05) and CRP (OR: 2.54; 95% CI: 1.25–5.17; *p*-value = 0.01) during the 6–18 months of follow-up. CRP mean values were above the normal range (>3.0 mg/L) during follow-up (month-12: 3.7 mg/L vs. 2.4 mg/L, *p* < 0.001; month-18: 3.7 mg/L vs. 2.7 mg/L, *p* < 0.001; Supplement, Section S3, Table S4). A similar result was observed in patients reporting RESc compared to controls (lymphocytes: OR: 1.35; 95% CI: 0.99–1.83; *p*-value = 0.06; CRP: OR: 1.21; 95% CI: 1.08–1.36; *p*-value < 0.01), with mean CRP levels above the normal range at each of the time-points (month 6: 3.6 mg/L vs. 2.7 mg/L, *p* < 0.001; month 12: 4.3 mg/L vs. 2.6 mg/L, *p* < 0.001; month 18: 4.4 mg/L vs. 2.8 mg/L, *p* < 0.001; Supplement, Section S3, Table S5). Patients with CFc, CPc, and NSc did not present a significant alteration in biochemical parameters compared to controls at multivariable analysis. The analysis of changes in the odds of developing PCC for every possible biochemistry value showed that patients with increasing CRP levels present progressively higher odds of falling within the respiratory cluster during follow-up (Supplement, Section S1, Figure S2). The same result holds for CRP and lymphocyte changes in PCC (Supplement, Section S1, Figures S3 and S4).

#### 3.3.3. Analysis of the Impact of the Severity of Acute COVID-19 Infection on Biochemical Features Measured During Follow-Up Among Patients with PCC

**Figure 2:**
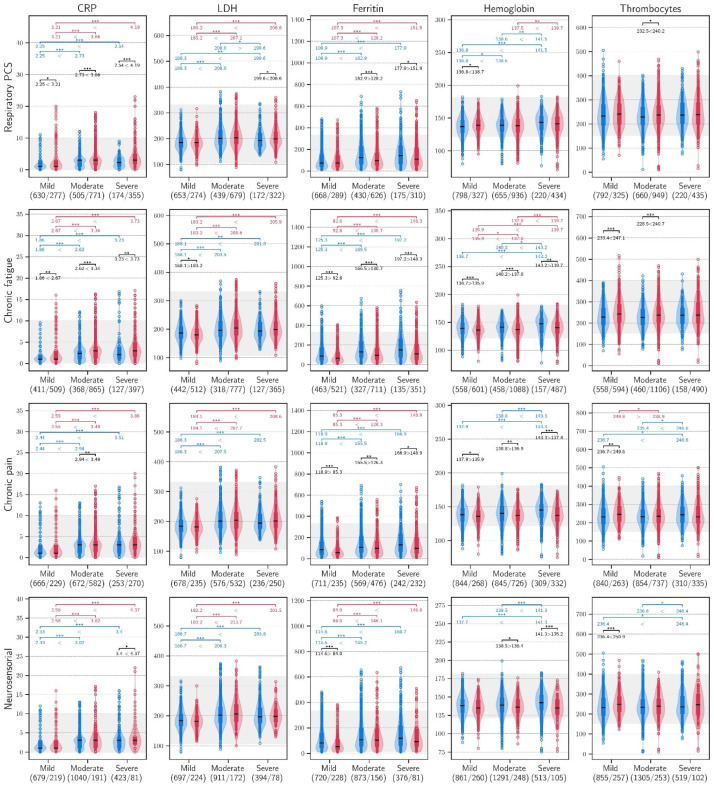
C-reactive protein, lactate dehydrogenase, ferritin, haemoglobin, and thrombocyte values during follow-up in patients within the four clusters of PCC, stratified according to the severity of the acute infection (blue: non PCC; red: PCC). AST: aspartate aminotransferase (U/L); LDH: lactate dehydrogenase (U/L); *** ≤ 0.001, ** ≤ 0.010, * ≤ 0.050.

Among patients with PCC, the CRP, LDH, and ferritin values measured during follow-up were higher in the patients who had experienced a moderate or severe acute infection compared to those who had a mild acute COVID-19 infection (*p* < 0.001), across all PCC clusters (Figure 2).

## 4. Discussion

The present paper reports the results of a robust statistical analysis to assess the role of routine biochemical tests, including full blood count, liver and renal function tests, CRP, LDH, ferritin, and blood glucose, in the acute phase of SARS-CoV-2 infection to predict both PCC occurrence and its specific presentation during follow-up. By accounting for all the possible confounders, an innovative statistical approach was able to capture the relevant associations, thus facilitating the interpretation of the results and improving the understanding of specific biological mechanisms leading to PCC in our cohort. The stratification of the analysis according to the PCC clusters identified through a PCA further enhanced the specificity of the findings, leading to a better characterisation of PCC in its different phenotypes. Through this approach, this study demonstrates that CRP levels are increased from 6- to 18-months post-acute infection in patients experiencing PCC overall and in those with respiratory sequelae. Furthermore, the analysis of laboratory parameters during follow-up in patients with PCC revealed that CRP values above 3 mg/L fall within the range observed in sustained and prolonged low-grade inflammation (3–10 mg/L), that elevated CRP levels measured during follow-up are directly proportional to PCC probability, and that this association is independent of confounders, including age, sex, comorbidities, and acute-phase treatment. In our cohort, an association between the severity of the acute COVID-19 infection and the alteration of CRP, ferritin and LDH values during follow-up was also found in patients with PCC but not in patients without. Finally, this study highlights that biochemical abnormalities detected during the early stages of acute COVID-19 do not correlate with an increased risk of developing PCC during follow-up.

Several reports and systematic reviews compared biochemical values among patients with PCC and individuals who fully recovered after the acute infection, finding differences in the expression of inflammation and vascular biomarkers [34]. However, most of them focused on laboratory tests that are not commonly assessed in routine clinical practice, such as pro-inflammatory cytokines. Hence, despite their unquestioned relevance from a scientific point of view, doubts arise about the possibility of using them to evaluate patients at risk of PCC or as a tool to monitor recovery during follow-up. When routine laboratory tests were assessed, higher levels of inflammatory biomarkers and coagulation parameters were detected [35,36]. However, in large prospective cohort studies with a long-term follow-up, none of those tests revealed a clear and meaningful association with PCC [37]. Furthermore, mean values of the laboratory parameters are often within normal range, thus questioning the clinical utility of the findings [38]. CRP is a well-known cytokine-mediated acute-phase reactant with recognised diagnostic importance in a wide range of diseases [39], but with controversial clinical value in the context of PCC [40]. Several studies available in the literature found differences between mean CRP serum levels in patients with PCC compared to healthy controls or patients who recovered after initial PCC [34,35,36]. However, the duration of follow-up is often limited and a cross-sectional design is predominant, thus hampering the possibility to interpret results across time. Furthermore, the non-linear progression of PCC over time can limit the capacity of a laboratory-based follow-up with fixed time points to detect biochemical fluctuation. It is widely agreed that CRP values may vary across laboratories; however, a value lower than 3 mg/L is generally considered normal, while 3–10 mg/L corresponds to a minor elevation, as observed in cases of low-grade inflammation, defined as a chronic, ineffective inflammatory state that leads to oxidative stress and tissue damage [41,42,43,44]. In our cohort, an independent significant difference in mean CRP serum levels was observed between patients with and without PCC and RESc, corresponding to low-grade inflammation during multiple time points up to 18 months after acute infection. Although not significant in multivariable analysis, CRP levels above 3 mg/L were also observed in the CF and CP clusters and in severe PCC across the follow-up time points, suggesting a sustained and prolonged inflammation among patients experiencing the persistence of multiple PCC symptoms. Several research groups have proposed that PCC is due to multifactorial processes including alterations in immune-inflammatory response and oxidative stress involving cytokine production and ongoing chronic inflammation [45]. Indeed, low-grade chronic inflammation presents with many symptoms that are usually reported in the context of PCC, such as chronic fatigue, post-exertional malaise, chronic pain, mild dyspnoea, and neurological symptoms [16]. Accordingly, CRP elevation within the range of low-grade chronic inflammation represents a biochemical marker of PCC that can be used both to support a clinical suspicion and to track PCC evolution over time. It has been proposed that acute COVID-19 could evolve into life-threatening systemic inflammation [46], while PCC can be the result of a less intense but prolonged inflammatory response [40]. In the present cohort, there is an association between the severity of acute COVID-19 and the risk of developing PCC, but no clear correlation between acute laboratory abnormalities and the risk of PCC has been demonstrated. Conversely, in patients with PCC and a previous moderate or severe acute infection, increased mean serum levels of CRP, ferritin, and LDH were detected during follow-up, suggesting that persistent inflammation and the severity of acute COVID-19 are related. These findings highlight the complexity of the pathogenesis of PCC, whose biological mechanisms are not yet fully understood.

This study presents some limitations. First, patients with a higher burden of PCC symptoms were more likely to adhere to the follow-up proposed. This could have introduced a selection bias in our cohort. Regarding the data collected prospectively, information on imaging and other tests (i.e., spirometry and cardiac ultrasound) were also recorded but exhibited a high percentage of missing or non-standardised information, thus hampering the possibility to analyse these features across the multiple centres included in this study. A high proportion of missing information was also observed among several biochemical markers (Supplement, Section S1, Figure S5). Given that multiple imputation can introduce bias when missing data exceeds certain thresholds (e.g., usually 5% [47]), we opted to exclude variables with more than 60% missing values and performed a complete-case analysis. However, while complete-case analysis may reduce potential imputation bias, it also reduces the sample size and may introduce bias if the missing values are not completely at random (MCAR). Future studies with more complete data collection would help validate these findings. The use of the two-step procedure, in which we first selected predictors from a regularised logistic or GLMM model, followed by a second step, in which we fit a non-penalised model to estimate confidence intervals and *p*-values, could have introduced bias. Although this approach is widely adopted in biomedical research to facilitate inference [48], it does not account for the uncertainty introduced by the variable selection step. Future work may consider more advanced methods for post-selection inference to mitigate these issues.

In conclusion, our findings indicate that, in our cohort, persistent low-grade systemic inflammation, reflected by sustained CRP elevation, represents a robust biochemical indicator of PCC, particularly in patients with respiratory sequelae and in those who experienced more severe acute infection. Although routine laboratory abnormalities during the acute phase did not predict PCC in our study, longitudinal CRP monitoring provides clinically relevant insight into its persistence and phenotype. These results support the hypothesis that PCC arises, at least in part, from maladaptive immune-inflammatory responses extending beyond the acute infection. From a clinical perspective, the recognition of low-grade inflammation as a hallmark of PCC underscores the potential utility of conventional laboratory markers both for patient monitoring and as endpoints in interventional studies. Future research should clarify the value of inflammation in PCC, exploring targeted anti-inflammatory strategies and integrative, symptom-tailored approaches to improve long-term outcomes in affected individuals.

## Figures and Tables

**Table 1:** Epidemiological and demographic characteristics of PCC and its clusters.

	PCC	RESc	NSc	CPc	CFc
**Demographic**					
Total number of patients	947	940	1008	973	890
Age, N (SD)	55.2 ± 14.3	55.2 ± 14.3	55.3 ± 14.3	55.1 ± 14.3	55.1 ± 14.3
Female, N (% *)	504 (8.9%)	501 (53.3%)	526 (52.2%)	519 (53.3%)	476 (53.5%)
**Underlying medical conditions**	N (% *)	N (% *)	N (% *)	N (% *)	N (% *)
Smoker	78 (8.9%)	78 (8.9%)	83 (8.8%)	81 (8.9%)	74 (9%)
Pregnancy	3 (0.6%)	3 (0.6%)	5 (1%)	5 (1%)	3 (0.7%)
Diabetes	2 (0.2%)	2 (0.2%)	2 (0.2%)	2 (0.2%)	2 (0.2%)
HIV ^1^	9 (1%)	9 (1%)	10 (1%)	10 (1%)	9 (1%)
Transplant recipients	10 (1.1%)	11 (1.2%)	11 (1.1%)	11 (1.1%)	9 (1%)
Auto-inflammatory Disease ^2^	55 (5.8%)	54 (5.7%)	58 (5.8%)	58 (6%)	53 (6%)
Cardiovascular Disease ^3^	379 (40.2%)	376 (40.1%)	406 (40.3%)	387 (40%)	354 (40%)
Chronic Liver Disease ^4^	27 (2.9%)	27 (2.9%)	28 (2.8%)	28 (2.9%)	25 (2.8%)
Chronic Kidney Disease ^5^	26 (2.8%)	27 (2.9%)	28 (2.8)	27 (2.8%)	24 (2.8%)
Obesity	60 (6.3%)	60 (6.4%)	65 (6.4)	64 (6.6%)	58 (6.5%)
Chronic Respiratory Disease ^6^	137 (14.5%)	137 (14.6%)	148 (14.7%)	145 (15%)	126 (14.2%)
Neurological Disorders	40 (4.2%)	40 (4.3%)	39 (3.9)	40 (4.1%)	39 (4.4%)
**Acute Infection**	N (% *)	N (% *)	N (% *)	N (% *)	N (% *)
Vaccination before Acute Infection	166 (23.5%)	166 (23.6%)	170 (22.6%)	167 (22.9%)	161 (24.7%)
First Wave ^7^	178 (18.8%)	175 (18.5%)	198 (19.6%)	183 (18.8%)	141 (15.8%)
Second Wave ^8^	223 (23.5%)	221 (23.5%)	234 (23.2%)	229 (23.5%)	217 (24.4%)
Third Wave ^9^	280 (29.6%)	280 (29.8%)	299 (29.7%)	291 (29.9%)	270 (30.3%)
Fourth Wave ^10^	157 (16.6%)	155 (16.5%)	162 (16.1%)	158 (16.2%)	154 (17.3%)
Hospital Admission	389 (41.1%)	388 (41.3%)	427 (42.4%)	408 (41.9%)	341 (38.3%)
Intensive Care Unit Transfer	91 (9.6%)	90 (9.6%)	102 (10.1%)	91 (9.4%)	80 (9%)
Overall Oxygen Therapy	325 (34.3%)	323 (34.4%)	349 (35.2%)	339 (35%)	285 (32.2%)
Corticosteroids Administration	379 (40.2%)	375 (39.9%)	397 (40.4%)	394 (40.5%)	359 (40.3%)
Ambulatory Mild Disease ^11^	535 (69.4%)	529 (69.2%)	555 (68.3%)	540 (68.8%)	529 (70%)
Hospitalised: Moderate Disease ^11^	132 (17.1%)	132 (17.3%)	141 (17.3%)	107 (13.6%)	127 (16.8%)
Hospitalised: Severe Diseases ^11^	104 (13.5%)	103 (13.5%)	117 (14.4%)	188 (19.3%)	100 (13.2%)

* Percentages were calculated on the total number of records without missing values; PCC: post-COVID condition, RES: respiratory cluster, NSc: neurosensorial cluster, CPc: chronic pain cluster, CFc: chronic fatigue cluster; ^1^ Human Immunodeficiency Virus ^2^ rheumatic disease, inflammatory bowel disease (ulcerative colitis, Crohn’s disease), psoriatic rheumatism, autoimmune hepatitis, psoriasis, atopic dermatitis, chronic urticarial, multiple sclerosis, inflammatory myopathy, systemic lupus erythematosus, systemic scleroderma, Sjögren’s syndrome, Behcet’s syndrome, atrophic polychondritis, antiphospholipid syndrome, Takayasu arteritis, Horton’s disease, knotty periarthritis, Kawasaki’s disease, microscopic polyangiitis, Wegener’s disease, Churg–Strauss syndrome, rheumatoid purpura, Buerger’s disease, cryoglobulinemia, sarcoidosis; ^3^ hypertension, congestive heart failure, coronary heart disease; ^4^ chronic liver disease other than cancer, including alcohol-induced liver disease, non-alcoholic fatty liver disease, non-alcoholic steatohepatitis, autoimmune hepatitis, primary biliary cirrhosis, hereditary hemochromatosis, Wilsons’ disease; ^5^ kidney damage for >3 months; ^6^ asthma, chronic obstructive pulmonary disease, obstructive sleep apnoea syndrome, pulmonary hypertension, restrictive lung disease. ^7^ First wave: SARS-CoV-2 infection occurring before 31.08.2020. ^8^ Second wave: SARS-CoV-2 infection occurring between 1.09.2020 and 31.12.2020. ^9^ Third wave: SARS-CoV-2 infection occurring between 1.01.2021 and 31.08.2021. ^10^ Fourth wave: SARS-CoV-2 infection occurring after 1.09.2021. ^11^ According to the WHO Clinical Progression Scale [33].

**Table 2:** Multivariable model of features, including biochemical parameters, associated with PCC and its four clinical clusters between 6 and 18 months of follow-up.

	PCC (N = 947)				RESc (N = 940)				CFc (N = 890)				CPc (N = 973)				NSc (N = 1008)			
	OR	LB	HB	*p*	OR	LB	HB	*p*	OR	LB	HB	*p*	OR	LB	HB	*p*	OR	LB	HB	*p*
INSERM									1.48	0.87	2.52	0.15	1.91	0.98	3.74	0.06	0.40	0.04	4.17	0.44
SAS	1.41	0.74	2.68	0.30	2.98	1.87	4.76	<0.01												
UNIVR	0.88	0.57	1.38	0.58									0.57	0.34	0.98	0.04	1.69	0.93	3.07	0.09
Age 31–40																				
Age 41–60					1.45	1.06	1.97	0.02					1.57	1.08	2.29	0.02	0.59	0.35	0.99	0.05
Age 61–80	0.73	0.54	0.99	0.04													0.51	0.27	0.98	0.04
Age > 80	0.38	0.18	0.83	0.02									0.65	0.20	2.11	0.48				
Female sex	1.70	1.28	2.26	<0.01					2.31	1.65	3.24	<0.01	2.68	1.78	4.04	<0.01	1.73	1.08	2.77	0.02
Chronic resp diseases					1.74	1.16	2.59	<0.01												
Cardiovascular diseases																	0.79	0.49	1.28	0.34
Obesity																				
Previous smoking																	0.69	0.41	1.18	0.18
Smoking																	1.14	0.57	2.29	0.72
First wave ^1^																				
Second wave ^2^																	1.80	1.14	2.84	0.01
Fourth wave ^3^									1.44	0.93	2.22	0.10								
Corticosteroid	1.44	1.07	1.95	0.02	1.26	0.88	1.81	0.20	1.47	1.09	2.00	0.01								
Monoclonal antibodies	0.56	0.39	0.81	<0.01					0.61	0.40	0.94	0.02	0.54	0.32	0.94	0.03				
Azithromycin																				
Oxygen therapy					1.49	1.01	2.20	0.04												
Renal events	3.45	0.98	12.1	0.05	2.31	0.76	6.99	0.14	3.14	1.02	9.70	0.05								
Pulmonary events	0.24	0.09	0.67	<0.01																
Gastro events									1.17	0.86	1.59	0.31								
Cardiac events																				
Other events																	0.61	0.23	1.66	0.34
ICU admission					1.43	0.85	2.42	0.18									0.76	0.32	1.78	0.52
General symptoms	1.98	1.06	3.70	0.03					8.97	2.65	30.41	<0.01								
Resp symptoms					2.44	1.42	4.19	<0.01									0.71	0.40	1.26	0.24
Gastro symptoms																	0.67	0.43	1.06	0.09
Neuro symptoms	2.01	1.47	2.74	<0.01					1.78	1.28	2.46	<0.01	1.31	0.84	2.02	0.23	9.74	4.88	19.43	<0.01
CRP	2.54	1.25	5.17	0.01	1.21	1.08	1.36	<0.01												
Haemoglobin									2.96	0.77	11.37	0.11	1.77	0.46	6.82	0.41				
Ferritin																				
LDH	0.19	0.02	1.61	0.13																
AST																	0.81	0.03	19.67	0.89
ALT																	0.26	0.03	2.63	0.25
Creatinine													1.13	0.85	1.49	0.40				
Leukocytes																				
Neutrophils																	2.61	0.60	11.42	0.20
Lymphocytes	3.12	1.05	9.28	0.05	1.35	0.99	1.83	0.06	2.04	0.80	5.22	0.14								
Platelets	1.54	0.41	5.83	0.53					2.95	0.76	11.55	0.12								

^1^ First wave: SARS-CoV-2 infection occurring before 31.08.2020; ^2^ second wave: SARS-CoV-2 infection occurring between 1.09.2020 and 31.12.2020; ^3^ fourth wave: SARS-CoV-2 infection occurring after 1.09.2021. The red font color indicates statistical significant covariates.

## Data Availability

Original data are unavailable due to privacy restriction; cumulative data are available at the project website: https://orchestra-cohort.eu/.

## Supplementary Materials

The following supporting information can be downloaded at https://www.mdpi.com/article/10.3390/biomedicines14010083/s1, Figure S1: Results of the PCA analysis; Figure S2: Odds ratios for C-reactive protein; Figure S3: Odds ratios for lymphocytes; Figure S4: Odds ratios for C-reactive protein (CRP); Figure S5: Average percentage of missingnes by variable and PCC phenotypes used for filtring; Table S1: Definitions; Table S2: Summary of the WP2 ORCHESTRA cohorts and ethic approvals; Table S3: Schedule of follow-up; Table S4: Univariable analysis of biochemical parameters associated with the occurrence of PCC divided per time point (only significant association are showed); Table S5: Univariable analysis of biochemical parameters associated with the occurrence of respiratory cluster of PCC divided per time point (only significant association are showed); Table S6: Univariable analysis of biochemical parameters associated with the occurrence of chronic fatigue cluster of PCC divided per time point (only significant association are showed); Table S7: Univariable analysis of biochemical parameters associated with the occurrence of chronic pain cluster of PCC divided per time point (only significant association are showed; Table S8: Univariable analysis of biochemical parameters associated with the occurrence of neurosensorial cluster of PCC divided per time point (only significant association are showed; Table S9: Univariable analysis of biochemical parameters associated with the occurrence of severe PCC divided per time point (only significant association are showed; Table S10: Epidemiological and demographic characteristics of panel subsamples selected for the multivariable analysis of the impact of biochemical parameters measured during the acute infection on the development of PCC and its clinical clusters; Table S11: Epidemiological and demographic characteristics of panel subsamples selected for the multivariable analysis of the impact of biochemical parameters measured during the follow-up on the development of PCC and its clinical cluster; Table S12: Multivariable model including biochemical parameters measured during the acute infection correlated with the occurrence of PCC and the distinct clusters.
