# Supplementary material for: Persistent Low-Grade Inflammation and Post-COVID Condition: Evidence from the ORCHESTRA Cohort
Source: Biomedicines. 2025 Dec 31;14(1):83. doi: 10.3390/biomedicines14010083 (PMC12839268; doi:10.3390/biomedicines14010083)
Supplement: Supplementary file 1 [file biomedicines-14-00083-s001.zip › biomedicines-3997284-supplementary.pdf]

Article

Persistent Low-Grade Inflammation and Post-COVID Condition: Evidence from the ORCHESTRA Cohort

Elisa Gentilotti [1,*], Carolina Alvarez Garavito [2,3], Anna Górska [1], Roy Gusinow [2,3], Lorenzo Maria Canziani [1], Pasquale De Nardo [1], Alessandro Visentin [1], Maria Giulia Caponcello [4,5], Michela Di Chiara [6], Aline-Marie Florence [7,8], Gerolf de Boer [9], Salvatore Cataudella [10], the ORCHESTRA Study Group [†], Gabriel Levy Hara [11], Adriana Tami [9], Maddalena Giannella [6,12], Cédric Laouénan [7,8], Jan Hasenauer [2,3], Jesús Rodríguez-Baño [4,5] and Evelina Tacconelli [1]

[1] Infectious Diseases, Department of Diagnostics and Public Health, University of Verona, 37129 Verona, Italy; anna.gorska@univr.it (A.G.); lorenzomaria.canziani@univr.it (L.M.C.); pasquale.denardo@univr.it (P.D.N.); alessandro.visentin@univr.it (A.V.); evelina.tacconelli@univr.it (E.T.)

[2] Life and Medical Sciences (LIMES), University of Bonn, 53113 Bonn, Germany; carolina.alvarez@uni-bonn.de (C.A.G.); roy.gusinow@uni-bonn.de (R.G.); jan.hasenauer@uni-bonn.de (J.H.)

[3] Bonn Center for Mathematical Life Sciences, University of Bonn, 53113 Bonn, Germany

[4] Infectious Diseases and Microbiology Unit, Hospital Universitario Virgen Macarena and Department of Medicine, University of Sevilla/Biomedicines Institute of Sevilla, CSIC, 41009 Sevilla, Spain; gcaponcello@gmail.com (M.G.C.); jesusrb@us.es (J.R.-B.)

[5] CIBERINFEC, Instituto de Salud Carlos III, 28029 Madrid, Spain

[6] Infectious Diseases Unit, Department for Integrated Infectious Risk Management, IRCCS Azienda Ospedaliero-Universitaria di Bologna, 40138 Bologna, Italy; michela.dichiara@aosp.bo.it (M.D.C.); maddalena.giannella@unibo.it (M.G.)

[7] Université Paris Cité, INSERM IAME UMR 1137, 75018 Paris, France; aline-marie.florence@aphp.fr (A.-M.F.); cedric.laouenan@inserm.fr (C.L.)

[8] AP-HP Nord, Hôpital Bichat, Department of Epidemiology Biostatistics and Clinical Research, 75018 Paris, France

[9] University Medical Center Groningen, Department of Medical Microbiology, and Infection Prevention, University of Groningen, 9713 GZ Groningen, The Netherlands; g.c.de.boer@umcg.nl (G.d.B.); a.tami@umcg.nl (A.T.)

[10] CINECA Interuniversity Consortium, 40126 Bologna, Italy; s.cataudella@cineca.it

[11] Instituto Alberto Tachini, School of Medicine, University of Buenos Aires, Buenos Aires C1123AAR, Argentina; educacionmedica.api@gmail.com

[12] Department of Medical and Surgical Sciences, Alma Mater Studiorum University of Bologna, 40138 Bologna, Italy

* Correspondence: elisa.gentilotti@univr.it

† The ORCHESTRA Study Group is listed in the Supplementary Materials.

TABLE OF CONTENTS

SECTION S1: METHODS 4

Study design 4

Objectives 4

Inclusion criteria 4

Exclusion criteria 4

Enrolment and structure of the follow-up 4

Data collection 5

Assessment of the quality of life through SF-36 questionnaire 6

Principal Component Analysis (PCA) 6

Figure S1. Results of the PCA analysis 7

a) Variance explained by PCA components 7

b) Loading matrix visualisation for the first three components 8

Statistical analysis 9

Filtering, normalisation and transformation 9

OR for changes in biochemical markers 11

Interpretation of C-reactive protein and lymphocytes results for multivariable models of biochemical parameters correlated with PCC and the clinical clusters between 6- and 18-months of follow-up 12

Figure S2. Odds ratios for C-reactive protein (CRP) 12

Figure S3. Odds ratios for lymphocytes 12

Figure S4. Odds ratios for C-reactive protein (CRP) 13

Figure S5. Average percentage of missingnes by variable and PCC phenotypes used for filtring 14

SECTION S2: DESCRIPTION OF THE COHORT AND ENROLMENT PROCEDURES 15

Table S1. Definitions 15

Table S2. Summary of the WP2 ORCHESTRA cohorts and ethic approvals 19

Table S3. Schedule of follow-up 20

SECTION S3: ADDITIONAL RESULTS 24

Table S4. Univariable analysis of biochemical parameters associated with the occurrence of PCC divided per time point (only significant association are shown). 24

Table S5. Univariable analysis of biochemical parameters associated with the occurrence of respiratory cluster of PCC divided per time point (only significant association are shown). 25

Table S6. Univariable analysis of biochemical parameters associated with the occurrence of chronic fatigue cluster of PCC divided per time point (only significant association are shown). 26

Table S7. Univariable analysis of biochemical parameters associated with the occurrence of chronic pain cluster of PCC divided per time point (only significant association are shown). 28

Table S8. Univariable analysis of biochemical parameters associated with the occurrence of neurosensorial cluster of PCC divided per time point (only significant association are shown). 29

Table S9. Univariable analysis of biochemical parameters associated with the occurrence of severe PCC divided per time point (only significant association are shown). 30

Table S10. Epidemiological and demographic characteristics of panel subsamples selected for the multivariable analysis of the impact of biochemical parameters measured during the acute infection on the development of PCC and its clinical clusters... 31

Table S11. Epidemiological and demographic characteristics of panel subsamples selected for the multivariable analysis of the impact of biochemical parameters measured during the follow-up on the development of PCC and its clinical clusters 33

Table S12. Multivariable model including biochemical parameters measured during the acute infection correlated with the occurrence of PCC and the distinct clusters..................................... 35

SECTION S1: METHODS

Study design

The ORCHESTRA WP2 includes six prospective cohorts (56 centres) from five countries (France, Italy, Netherlands, Spain, and Argentina) with laboratory-confirmed SARS-CoV-2 infection between February 2020 and December 2021 and followed up at 3, 6, and 12 months post-acute infection. For the present study, data were extracted on 27.10.2022 from the REDCap database, to include all of the patients with an available 12-month follow-up assessment by 30 June 2022, together with the information on acute infection.

This study is registered on ClinicalTrial.gov (ID: NCT05097677), and the protocol is available on the institution's website.

Objectives

The primary objectives of this study were as follows: to describe the prevalence, duration, and clustering of symptoms of post-COVID-19 condition (PCC); to investigate preventive and risk factors for PCC by symptom clusters, comorbidities, severity, and treatment of acute infection (including early treatments), vaccination status, VoC, and anti-S Ab titre; and to analyse the severity of PCC according to the impact of symptom clusters on patients' quality of life.

Inclusion criteria:

- Age >14 years old;
- Laboratory-confirmed SARS-CoV-2 infection;
- Written informed consent.

Exclusion criteria:

- Age < 14 years old;
- Clinical diagnosis of SARS-CoV-2 with no available laboratory confirmation;
- No written informed consent signed.

Enrolment and structure of the follow-up

Both patients requiring hospital admission during the acute infection and outpatients could be enrolled. Patients were enrolled during the acute infection (baseline) or at any of the subsequent

time points. In the first case, data about the acute infection were collected, respectively, while in the second case, they were retrieved retrospectively through patient interviews and medical records. The follow-up time points were the following: 3-, 6-, 12-, and 18-month post-infection. Each follow-up visit combined a clinical and laboratory assessment, as reported in table S3. A nasal swab was performed to identify the variant of concern (VOC) at baseline and repeated only if a sample tested positive more than 30 days after the initial infection diagnosis. VoC and serological analysis were performed at the Central Laboratory of Antwerp (SAS, UNIBO, and UNIVR) or at local laboratories (COVID Home, INSERM, and UBA) using homogenised protocols. Serological results in AU/ml were converted into BAU by multiplying with a seroconversion factor (1.0288 for Roche Elecsys Anti-SARS-CoV-2; 0.142 for Alignity_Abbott; and 0.00901 for MSD assay). For the purpose of the analysis, immunological anti-S response was classified as <1500 BAU, 1500-5000 BAU, 5000-16000 BAU, and >16000 BAU.

Data collection

Study data were collected and managed using REDCap electronic data capture tools hosted at CINECA. REDCap (Research Electronic Data Capture) is a secure, web-based software platform designed to support data capture for research studies, providing 1) an intuitive interface for validated data capture; 2) audit trails for tracking data manipulation and export procedures; 3) automated export procedures for seamless data downloads to common statistical packages; and 4) procedures for data integration and interoperability with external sources. The variables underwent a process of homogenisation across the different cohorts and standardisation according to the protocol. Since the cohorts in France (French COVID and in the Netherlands (COVID-HOME) started before the ORCHESTRA project was financed, data from these two cohorts went through a post-data collection harmonisation process under the supervision of the Charité – Universitätsmedizin Berlin and transformation conducted by the Centre Informatique National de l'Enseignement Superieur (CINES).

Data collected at baseline included date of symptom onset and diagnosis, duration of symptoms, demographic characteristics, comorbidities, clinical presentation, treatment during the acute infection, hospitalisation, admission to ICU, and post-acute infection complications. Early treatment was defined as monoclonal antibodies administered during the study period (bamlanivimab, bamlanivimab/etesevimab, casirivimab/imdevimab), received within the first 5 days of onset of symptoms according to national recommendations (data available only for UNIVR

cohort). A symptom was associated with SARS-CoV-2 infection if it was newly diagnosed after the acute infection or if a significant worsening in its severity and/or presentation was registered after acute infection. Occurrence of new medical events, vital signs and physical examination, laboratory parameters and vaccination status were also collected at each time point (table S4). Quality checks of variables were run by CINECA and errors were reported back to the cohort's local teams for correction. Data imputation was performed only in case of data that could be derived by known information, such as availability of the vaccination (e.g., patients infected before the start of vaccination campaigns within each country were considered as not vaccinated).

Assessment of the quality of life through the SF-36 questionnaire

The quality of life was assessed through the 36-Item Short Form Survey (SF-36). The SF-36 questionnaire is composed of 36 items categorised into right scales: physical functioning, role limitations due to physical health, pain, general health, vitality, social functioning, role limitations due to emotional problems, and mental health. Each of the items has between two and six response levels. For each scale, the items were (re)codified, transformed, and aggregated into a scale ranging from 0 to 100. In case of missing information, if the respondent has answered at least 50% of the items within the scale, the scale average was imputed into the missing items. Once the score for each of the 8 scales was computed, these were aggregated into two main components: the physical component summary (PCS) and the mental component summary (MCS) based on population-representative weights computed via PCA. The questionnaires were scored using the PRO CoRE software developed by QualityMetrics, which applies US1998 norms. The definition of a suboptimal score was based on the 25th percentile of the distribution for patients not reporting symptoms at the 12-month assessment.

Principal Component Analysis (PCA)

Based on both frequency and completeness of available information across all the cohorts, we restricted the number of symptoms to the following nine: cough, dyspnoea, fatigue, memory loss, headache, ageusia, anosmia, myalgia and arthralgia. We utilised an unsupervised machine learning algorithm called principal component analysis (PCA) to identify groups of symptoms. These groups were later used as results in the univariable and multivariable logistic regression to examine factors associated with each of the identified clinical phenotypes and their impact on quality of life measured with the SF-36 questionnaire. To reduce the number of symptoms, PCA

was utilised as a tool for dimensionality reduction. We created new dimensions based on linear combinations of the original variables that captured the highest variation while maintaining orthogonality between any two principal components.

Figure S1 a) graph shows the percentage variance attributed to PCA components corresponding to the nine largest eigenvalues. The first three components are essential, as they have corresponding eigenvalues that are more significant than one and explain around 54% of the total variance of the dataset. Figure S1 b) presents a matrix showing the oblique-rotated loading of the first three principal components. According to this matrix, anosmia/ageusia, and arthralgia/myalgia have larger loading values and contribute more to the second and third components, respectively. The third cluster was divided into two based on clinical significance to better understand the various symptoms associated with cough, dyspnoea, fatigue, headache, and memory loss. This resulted in four distinct clinical phenotypes: chronic pain (CPc), neurosensorial (NSc), respiratory (Res), and chronic fatigue (CF).

Figure S1. Results of the PCA analysis.

a) Variance explained by PCA components.

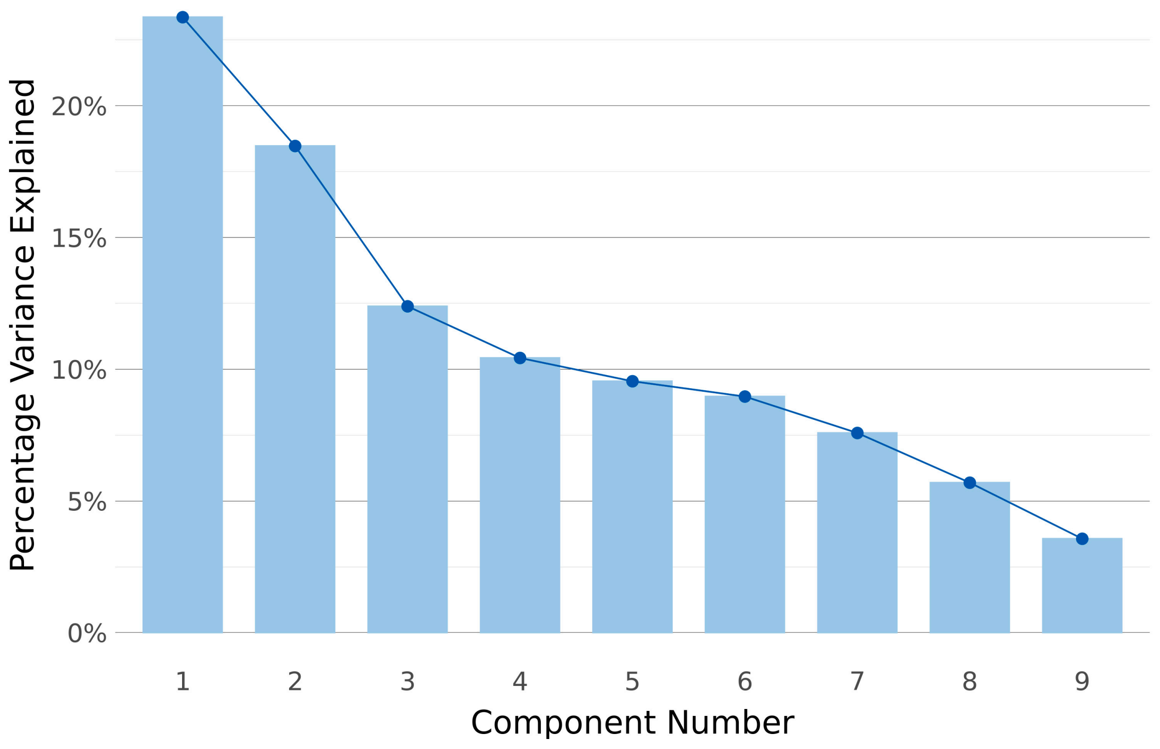

b) Loading matrix visualisation for the first three components.

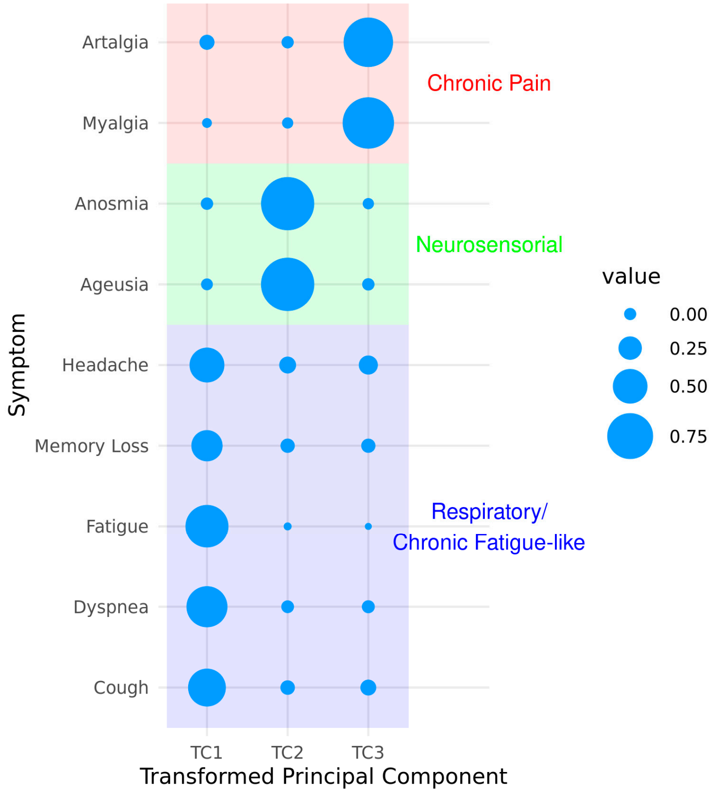

Statistical analysis

Filtering, normalisation, and transformation

Prior to fitting the multivariable models, we applied a systematic approach to select, filter, and prepare covariates, addressing the challenges posed by missing data and variable distributions. Initially, all covariates demonstrating statistically significant associations with any PCC phenotype at univariate analyses were considered, along with all available biochemical markers. Therefore, we excluded any covariate exhibiting over 60% missingness and proceeded with complete-case analyses rather than applying multiple imputation, given that imputation techniques become unreliable in case of missing data (supplement, figure S5) [28]. Biochemical markers that satisfied this filtering criterion were subsequently normalised against their clinical reference ranges, assigning a value of 0 to the lower bound and 1 to the upper bound. Values outside this normalised range were preserved, allowing the interpretation of values exceeding the normal limit; for example, a normalised value of 2 would represent a measurement double the upper reference limit. To further mitigate distributional skewness and stabilise variance, the normalised biochemical values were log10-transformed. To specifically avoid taking the logarithm of negative or zero values, we added a small, variable-specific constant—the absolute value of the minimum normalised value plus a small tolerance (1e-6)—to each marker before the transformation.

Longitudinal modelling of biochemical markers and PCC symptoms

Following Groll and Tutz (2014), we denote y_{it} as the outcome variable of patient i in time point, with time points ranging $t = 6,12,18$ months after acute infection. The outcome is a binary variable indicating whether a patient presents the symptoms associated with the . Statistically significant variables from univariate analysis are taken as possible confounders for the models, as well as all available biochemical variables. However, due to substantial missing data (particularly in biochemical markers), we excluded variables with over 60% missingness and conducted the analysis on complete-case only (i.e., a patient would be removed from the specific time point if variable is missing), as imputation methods are less reliable with high missingness. Included biochemical markers were normalised against clinical reference ranges, preserving values outside the normal limits. More formally, we define the normalised value as

$$X_{norm} = \frac{X - X_{min}}{X_{max} - X_{min}}, \quad (1)$$

where X_{min} and X_{max} refer to the lower and upper bounds of the clinical reference range for a variable, respectively. To stabilise variance and reduce skewness, a log-transformation was applied to the normalised biochemical variables. Since the normalisation procedure described above can result in negative values when $X_< X_{min}$, we added a variable-specific constant shift $s = |\min(X_{norm})| + \epsilon$ where $\epsilon = 10^{-6}$. Then, we constructed the log-transformed variable as

$$X_{log} = \log(X_{norm} + s) \quad (2)$$

After filtering and transformation, the covariate matrix for the fixed effects is denoted by X_{it}^T included variables measured only at acute phase such as demographic information, underlying medical conditions, acute-phase symptoms, hospitalisation status, cohort information, and COVID-19 clinical treatment, as well as normalised and log-transformed biochemical variables measured at 6, 12, and 18 months that met the inclusion criteria. We considered patient-specific random intercepts, which were included as random effects in the model. The resulting random-effects matrix is denoted Z_{it}^T, with the corresponding number of random effects specified below.

The longitudinal model was specified as

$$g(\mu_{it}) = X_{it,}^T \beta_+ Z_{it}^{\{T} \mathrm{b}_i \quad (3)$$

with the logit link $g(.)$ used to model the binary outcome. We use the glmmLasso() function from the glmmLasso R package developed by Groll and Tutz (2014) to fit an L_1-penalised regression to shrink the fixed effects coefficients for variable selection. For choosing the amount of regularisation in the model, we systematically explored the optimal amount of regularisation by evaluating a comprehensive sequence of λ values ranging logarithmically from 100 to 0.01, complemented by an additional evaluation at zero penalisation. The optimal model is chosen by minimising the Akaike Information Criterion (AIC) calculated at each λ step.

After determining the optimal regularisation strength, λ^*, we identify the active set of predictors as the subset of fixed-effect coefficients whose penalised estimates are non-zero

$$A = \{j: \widehat{\beta_J}(\lambda^*) \neq 0\} \quad (4)$$

where $\widehat{\beta_J}$ refers to the fixed effect coefficient of covariate jth of the penalised first stage. We re-fit a standard (non-penalised) generalised linear mixed-effects model using the glmer() R function from the R package lme4 using only the covariates selected using the active set A. The model specification is

$$g(\mu_{it}) = X_{it,A}^T \beta_A + Z_{it}^{\{T} \mathrm{b}_i, \quad (5)$$

where $X_{it,A}$represents the covariate matrix restricted to the variables selected in the penalisation process. This final model then reports standard errors and confidence intervals for all $\widehat{\beta_A}$.

Acute-to-follow-up predictive models

We conducted acute-to-follow-up analyses by implementing a similar approach to that used for the longitudinal models. Specifically, for each outcome y_i indicating the presence or absence of a specific PCC cluster at any time point between 6, 12, and 18 months after acute infection, we used the model

$$\log\big(P(y_i = 1)\big) = X_i^T \beta, \quad (6)$$

with X_i representing the covariate matrix comprising acute-phase demographic information, underlying medical conditions, acute-phase symptoms, hospitalisation status, cohort information, COVID-19 clinical treatment, and biochemical markers that were normalised and log-transformed. As with the longitudinal models, an inclusion criterion for covariates to be included in the model is that they do not present more than 60% of missing patient information. An L_1-penalised logistic regression was then fitted for the model specification using the same λ vector as previously described. The optimal regularisation strength λ^* was chosen by minimising the AIC. As with the longitudinal models, we then fit a reduced (non-penalised) logistic regression using only the active set of regressors identified at the optimal λ-step.

Odds Ratios for changes in biochemical markers

To help with the interpretation of the relationship between the biochemical markers identified as significant in our models and PCC, we performed a straightforward odds-ratio analysis based on changes in biochemistry levels. For each significant biomarker (e.g., CRP, lymphocytes), we defined a clinically relevant range of values commonly observed. We then considered every possible change from one biochemical value to another. Each change was represented by computing the numerical difference between the two biochemistry values after normalisation and log10-transformation, as previously described. Using these computed differences and the corresponding regression coefficients from our logistic regression models, we estimated odds ratios for each change. These odds ratios quantify how the risk of developing PCC symptoms changes when a biomarker moves from one specific level to another. Finally, we visualised these odds ratios in heatmaps, providing a clear depiction of the increase or decrease in risk associated with each biomarker level change.

Interpretation of C-reactive protein and lymphocytes results for multivariable models of biochemical parameters correlated with ~~post-COVID-19 condition~~ PCC and the clinical clusters between 6- and 18-months of follow-up.

To interpret how changes in biochemistry variables influence the risk of developing PCC or any of its phenotypes, we computed odds ratios (ORs) using the coefficients from the mixed effects regression models. Biochemistry variables were previously normalised and log-transformed, as described in Equation 2. We then computed the OR associated with a change between two biochemical levels (from an initial value X_{from} to a final value X_{to}) by exponentiating the product of the regression coefficient $\hat{\beta}_{GLMM}$ and the difference between the transformed values:

$$OR_{transition} = \exp[\hat{\beta}_{GLMM} \times (X_{to,log} - X_{from,log})] \quad (7)$$

The $OR_{transition}$ indicates the odds of developing PCC or a particular phenotype when the biochemistry variable increases or decreases from one clinical value to another.**2**

Figure S2. Odds ratios for C-reactive protein (CRP) change in respiratory cluster (RESc).

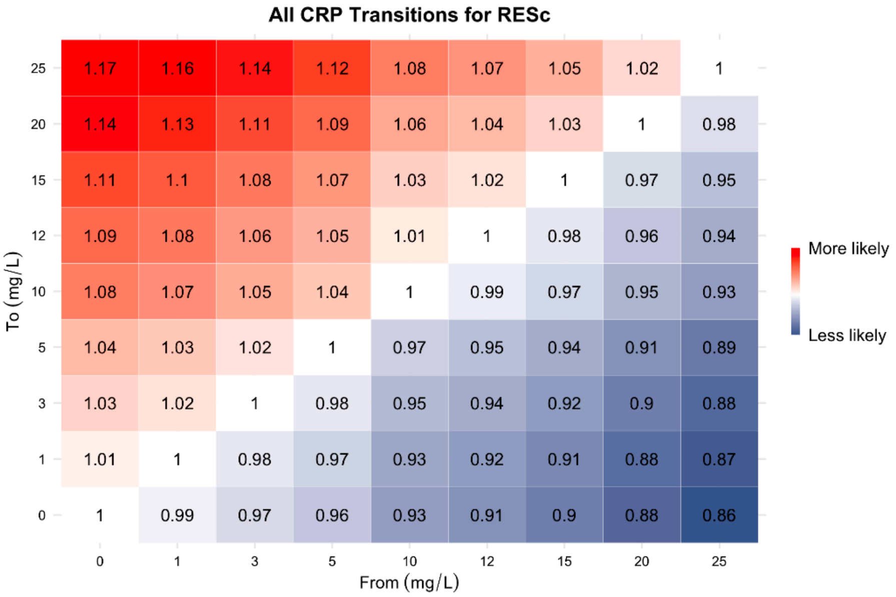

Figure S3. Odds ratios for lymphocyte change in PCC.

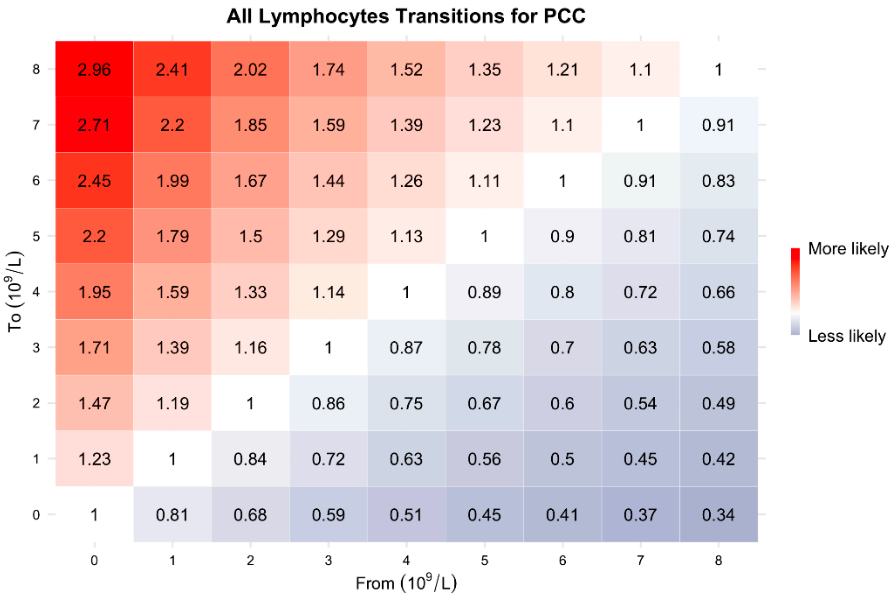

Figure S4. Odds ratios for C-reactive protein (CRP) change in PCC.

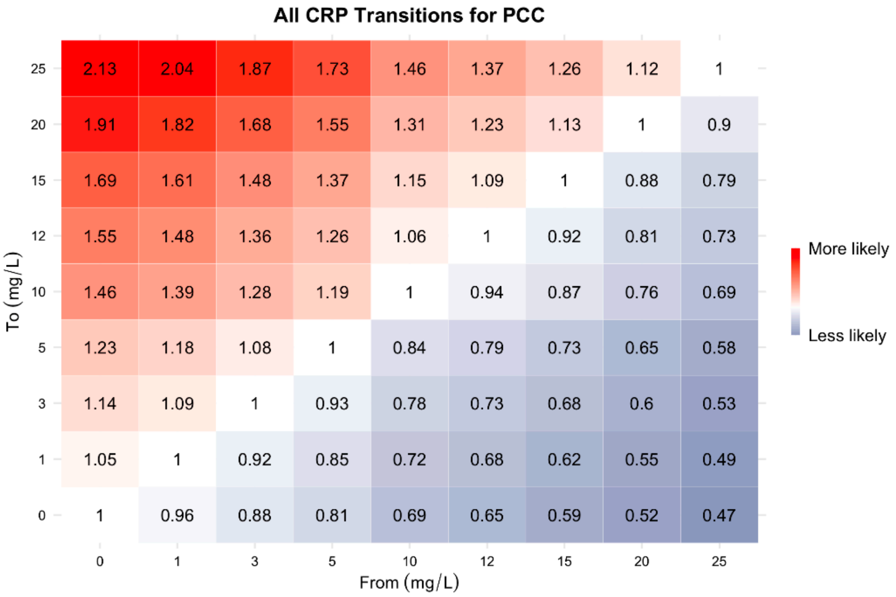

Figure S5. Average percentage of missingness by variable and PCC phenotypes used for filtering.

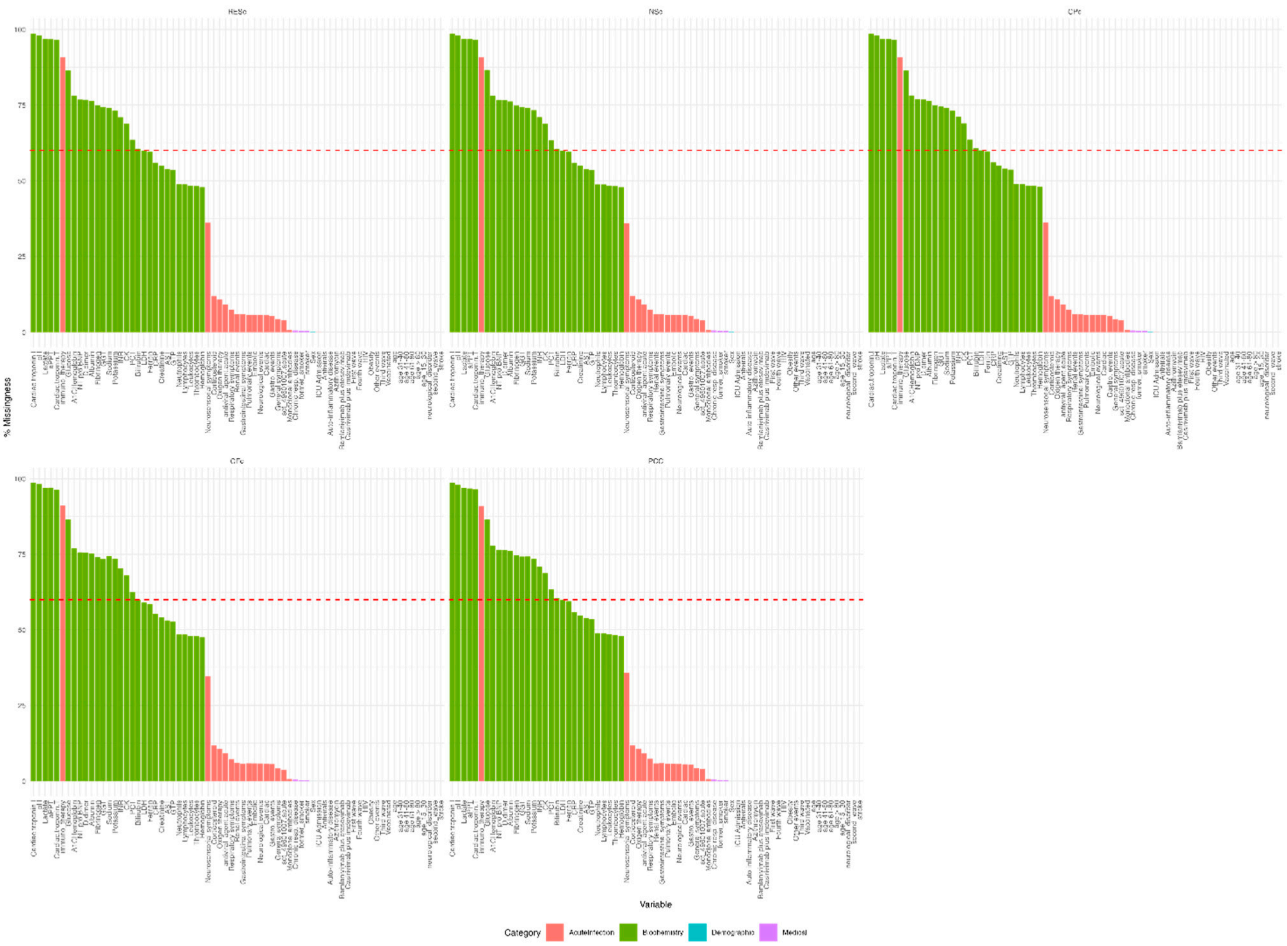

SECTION S2: DESCRIPTION OF THE COHORT AND ENROLMENT PROCEDURES

Table S1. Definitions

	Definition
**Cardiovascular disease**	Hypertension<br>Congestive heart failure<br>Coronary heart disease
**Chronic respiratory disease**	Asthma<br>Chronic obstructive pulmonary disease (COPD)<br>Obstructive sleep apnoea syndrome (OSAS)<br>Pulmonary hypertension<br>Restrictive lung disease
**Diabetes**	Diabetes type 1<br>Diabetes type 2
**Renal disease**	Kidney damage for > 3 months – includes:<br>Primary glomerular disease (focal segmental glomerulosclerosis, IgA nephropathy)<br>Secondary glomerular disease (diabetes with renal complications, systemic lupus erythematosus)<br>Tubulointerstitial diseases (sarcoidosis, drug-induced, urate, environmental toxins, myeloma)<br>Obstructive nephropathy<br>Vascular diseases (Atherosclerosis, hypertension, ischemia, cholesterol emboli, systemic vasculitis, thrombotic microangiopathy, systemic sclerosis)<br>Cystic and congenital diseases (Polycystic kidney disease, Alport's syndrome, Fabry's disease)
**Liver diseases**	Chronic liver disease other than cancer includes:<br>Alcohol induced liver disease<br>Non-alcoholic fatty liver disease (NAFLD)<br>Non-alcoholic steatohepatitis (NASH)<br>Autoimmune Hepatitis (AIH)<br>Primary Biliary Cirrhosis (PBC)<br>Hereditary Hemochromatosis<br>Wilsons' Disease
**Active cancer**	Solid tumour<br>Haematological malignancy
**Transplant recipients**	Solid organ<br>Bone marrow
**Autoimmune diseases**	Rheumatic disease

	Inflammatory bowel disease (ulcerative colitis, Crohn's disease)<br>Psoriatic rheumatism<br>Autoimmune hepatitis<br>Psoriasis<br>Atopic dermatitis<br>Chronic urticaria<br>Multiple sclerosis<br>Inflammatory myopathy<br>Systemic lupus erythematosus<br>Systemic scleroderma<br>Sjögren's syndrome<br>Behcet's syndrome<br>Atrophic polychondritis<br>Antiphospholipid syndrome<br>Takayasu arteritis<br>Horton disease<br>Polyarteritis nodosa<br>Kawasaki's disease<br>Microscopic polyangiitis<br>Wegener's disease<br>Churg-Strauss syndrome<br>Rheumatoid purpura<br>Buerger's disease<br>Cryoglobulinemia<br>Sarcoidosis
**Steroid therapy**	Dexamethasone<br>Prednisone<br>Methylprednisolone
**Anticoagulant therapy**	Unfractioned heparin<br>Low molecular weight heparin<br>Fondaparinux<br>Vitamin K antagonist (ex. Warfarin)<br>Direct oral anticoagulants (ex. dabigatran, rivaroxaban, apixaban, edoxaban, betrixaban)
**Antiviral therapy**	Ribavirin<br>Lopinavir/ritonavir<br>Interferon alpha<br>Interferon beta<br>Neuraminidase inhibitors<br>Favipiravir<br>Remdesivir<br>Camostat<br>Atazanavir<br>Darunavir
**Monoclonal antibody therapy**	Bamlanivimab<br>Bamlanivimab plus etesevimab

	Casirivimab plus imdevimab
**Immunomodulator therapy**	Tocilizumab<br>Sarilumab<br>Canakinumab<br>Siltuximab<br>Acalabrutinib<br>Ruxolitinib<br>Adalimumab<br>Etanercept<br>Baricitinib<br>Anakinra<br>Emapalumab<br>Cyclosporin A<br>Tacrolimus<br>Sirolimus<br>Everolimus
**General symptoms**	Fever<br>Fatigue<br>Myalgia<br>Arthralgia<br>Headache<br>Conjunctivitis<br>Lymphadenopathy<br>Anorexia<br>Skin rash<br>Haemorrhage
**Respiratory symptoms during acute infection**	Cough<br>Dyspnoea<br>Sore throat<br>Nasal congestion<br>Rhinorrhoea<br>Chest pain<br>Chest retraction<br>Wheezing
**Gastrointestinal symptoms during acute infection**	Abdominal pain<br>Diarrhoea<br>Nausea<br>Vomiting
**Neurological symptoms during acute infection**	Ageusia<br>Anosmia<br>Syncopal episodes<br>Confusion<br>Memory loss<br>Aphasia<br>Anomia<br>Seizures<br>Inability to walk

**Pulmonary complications**	Pulmonary aspergillosis<br>Pneumothorax<br>Pleural effusion<br>Cryptogenic organising pneumonia
**Cardiac complications**	Congestive heart failure<br>Cardiac arrhythmia<br>Myocarditis<br>Pericarditis<br>Cardiac ischemia<br>Cardiac arrest
**Embolic complications**	Pulmonary embolism<br>Deep vein thrombosis<br>Other thromboembolic events<br>Disseminated intravascular coagulation
**Neurological complications**	Meningitis<br>Encephalitis<br>Seizure<br>Stroke or cerebrovascular accident
**Renal complications**	Acute renal injury<br>Acute renal failure
**Gastrointestinal complications**	Gastrointestinal haemorrhage<br>Pancreatitis<br>Acute liver dysfunction

Table S2. Summary of the WP2 ORCHESTRA cohorts and ethic approvals

**Cohort**	**Ethical committee approval and enrolment start**
**University of Verona,** ***Italy***	13th April 2021
**University of Bologna UNIBO,** ***Italy***	3rd June 2021
**French Covid, Institut National de la Santé et de la Recherche Médicale,** ***France***	7th February 2020
**Andalusian Health Service,** ***Spain***	19th March 2020
**COVID-HOME, University Medical Center Groningen, the** ***Netherlands***	28th May 2021
**Universidad de Buenos Aires,** ***Argentina***	8th July 2021

Table S3. Schedule of follow-up.

	COVID-19 (2 weeks[1] ± 2 weeks)	3 months[1] ± 1 month	6 months[1] ± 1 month	12 months[1] ± 1 month	18 months[1] ± 2 months
**Screening/baseline**					
Inclusion criteria[1]					
Demographics[2]					
Healthcare setting[3]					
Length of hospital stay, days					
ICU admission					
Medical history[4]					
**Treatment**					
Comorbidity management[5]	X	X	X	X	X
Anti-COVID therapy[6]	X				
Antibiotic therapy[7]					
Oxygen therapy[8]	X	X*	X*	X*	X*
SARS-CoV-2 vaccination[9]	X	X	X	X	X
**Clinical assessment**					
Relevant medical new events[10]	X	X	X	X	X
COVID-19 symptom[11]onset	X				
COVID-19 symptom end	X	X*	X*	X*	X*
COVID severity[12]	X				
SOFA score	X				
Vital signs[13]	X	X	X	X	X
Physical examination[14]	X	X	X	X	X
12-lead electrocardiography	X	X	X	X	X
6-minute walking test	X	X	X	X	X
DLCO (diffusing capacity for carbon monoxide)	X	X	X	X	X
Pulmonary function test[15]	X	X	X	X	X
**Questionnaires**					
Functional status[16]	X	X	X	X	X
Respiratory impairment[17]	X	X	X	X	X
Mental health[18]	X	X	X	X	X
Perceived risk of re-infection/admission/re-admission[19]	X	X	X	X	X
Adherence to main preventative non-pharmacological measures[20]	X	X	X	X	X
SARS-CoV-2 vaccination: acceptance/non-acceptance and reasons[21]	X	X	X	X	X

**Imaging**					
Lung ultrasound	X	X	X*	X	X*
X-ray	X	X	X*	X*	X*
High-resolution CT scan	X	X	X*	X*	X*
Cardiac ultrasound	X	X	X*	X	X*
Cardiac MRI [22]	X[21]	X[21]	X*	X[21]	X*
**Biochemistry**					
Blood tests[23]	X	X	X*	X	X*
Arterial blood gas test (pO_2/pCO_2/pH)	X	X	X*	X*	X*
Urine tests[24]	X	X	X*	X	X*
**Immunology**					
N-IgG	X	X	X	X	X
N-IgM	X	X	X	X	X
N-IgA	X	X	X	X	X
S-IgG	X	X	X	X	X
S-IgM	X	X	X	X	X
S-IgA	X	X	X	X	X
**Microbiological tests**					
SARS-CoV-2 molecular test in nasopharyngeal swab or tracheal aspirate or bronchoalveolar lavage to detect the virus.	X	X*	X*	X*	X*
**Adjunctive variables for specific fragile populations**					
**HIV**					
HIV-infection status[25]	X	X	X	X	X
HIV-infection therapy[26]	X	X	X	X	X
Assessment of adherence to follow-up visits and antiretroviral therapy	X	X	X	X	X
**Elderly**					
Cognitive status[27]	X	X	X	X	X
**Pregnant women/new mother**					
History of positive SARS-CoV-2 molecular test on amniotic fluid or breast milk[28]					
History of detection of microthrombotic disease on placenta tissue or umbelical cord tissue					
**Children**					
History of positive SARS-CoV-2 molecular test on amniotic fluid or breast milk[29]					
Biometric paramethers[30]	X	X	X	X	X

**Transplant**					
Transplant general information[31]					
Graft function[32]	X	X	X	X	X
Immunosuppressive regimen[33]	X	X	X	X	X
**Onco-haematology**					
Assessment of adherence to oncologic follow-up visits and therapy	X	X	X	X	X
Assessment of progression of the disease and relapse	X	X	X	X	X
Assessment of adverse events[34]	X	X	X	X	X

Modular data capture according to level of commitment (level I, level II, level III).

Level I	Assessments in level I are mandatory
Level II	Customised according to the feasibility of each cohort

* Reassessed only if outside the normal ranges at the previous assessment or if clinically indicated

[1]Day 0: first positive SARS-CoV-2 test. Inclusion criteria for enrolment: inpatients and outpatients aged >14 years old with a laboratory-confirmed SARS-CoV-2 infection who signed the written informed consent. Exclusion criteria: age below 14 years, no written informed consent signed, and no SARS-CoV-2 test performed. [2]Demographics: age (years), sex, ethnic group (African, Asian, European, Latin America...), education (no formal education, lower than college, college or higher), cigarette smoking (never-smoker, former smoker, current smoker), usual residence (home, long-term care facility, public dormitory, prison, homeless), current occupation (student, unemployed with no benefits, unemployed with benefits, employed, self-employed, informal worker). [3]Healthcare setting: (a) outpatient (b) non-intensive care unit (c) intensive care unit. [4]Medical history: cardiovascular diseases (hypertension, coronary artery disease, congestive heart failure), diabetes (without insulin, with insulin), chronic respiratory disease (asthma, chronic obstructive pulmonary disease, obstructive sleep apnoea, restrictive lung disease, pulmonary hypertension), kidney disease (chronic with/without dialysis), liver disease other than cancer (HBV/HCV/HDV chronic viral hepatitis, other chronic disease, cirrhosis), metabolic disease, immunosuppressive conditions (solid organ transplant recipient, auto-immune diseases), cancer (solid cancer, haematological malignancies, type of primitive cancer/haematological malignancies, presence of metastases, if ongoing chemotherapy), mental or neurological disorders (psychiatric illness, anxiety disorder, mood disorder, psychotic disorder, Alzheimer disease, dementia other than Alzheimer, Parkinson's disease, myasthenia gravis, epilepsy, stroke (with/without residual deficits, neuromuscular disease, multiple sclerosis), muscular dystrophy, amyotrophic lateral sclerosis); TB co-infection; other opportunistic co-infection (specify) for HIV population. [5]Comorbidity management: drug name and dose (to include only treatments taken regularly). [6]Anti-COVID therapy: drug name, maintenance dose, and duration; [7]Antibiotic therapy: drug name, dose, duration, and type of treated infection. [8]Oxygen therapy: nasal prongs, face mask, face mask with reservoir, high-flow nasal cannula, non-invasive ventilation, mechanical ventilation; numbers of O_2 (L/min) provided (maximum reached) and fraction of inspired O_2 (FiO_2) provided (maximum reached). [9]SARS-CoV-2 vaccination: vaccine name, date of administration. [10]Relevant new medical events or worsening of previous conditions, including deep venous thrombosis, pulmonary embolism, infections (including a new SARS-CoV-2-infection during follow-up), malignancies (type of cancer, overall stage). [11]Symptoms: abdominal pain, ageusia/dysgeusia, anosmia, balance impairment, behaviour disorder, chest pain or chest tightness, confusion, cough, delirium, diarrhoea, disrupted sleep, dizziness, dyspnoea, fatigue, fever (including low-grade fever), headache, hypothermia, impaired cognitive status, lethargy, loss of appetite, mood affective disorder, myalgia, nausea/vomiting, palpitation, phlegm, runny nose, sore throat, stuffed nose, syncope, wheeze. [12]WHO Clinical Progression Scale. [13]Vital signs: dead/alive, blood pressure, body temperature, heart rate, respiratory rate, peripheral oxygen saturation. [14]Physical examination: BMI, abdominal examination, pulmonary examination, cardiac examination, neurological examination, peripheral vascular examination; [15]Pulmonary function test: FEV_1, FVC, FEV_1/FVC, TLC, FRC, RV. [16]Questionnaires to address the functional status: Post-COVID-19 Functional Status (PCFS) Scale, Global Physical Activity, Questionnaire (GPAQ), Barthel Index, Medical Outcome Study Short Form (MOS SF)-36 Score, EuroQol Five-Dimension Five-Level (EQ-5D-5L) questionnaire, Clinical Frailty Scale (CFS), Basic Activity of Daily Living (BADL). [17]Questionnaires to address the respiratory impairment: Saint George Respiratory Questionnaire (SGRQ), Transition Dyspnoea Index (TDI), mMRC (Modified Medical Research Council) Dyspnea Scale. [18]Questionnaires to address the mental health: Hospital Anxiety and Depression Scale (HADS), Kessler Psychological Distress Scale (K10), Impact of Event Scale—Revised (IES-R). [19]Perceived risk of re-infection on a scale 0-10 (no risk- very high risk); perceived risk of admission/re-admission on a scale 0-10 (no risk- very high risk). [20]Frequency of mask wearing (type of mask); frequency of hand washing; respect of social distance; avoidance of social gathering. [21]Was the vaccine accepted? Why was it not accepted (lack of trust in efficacy and/or safety; belief that it was not useful in the specific case; preference for someone else to receive it first)? [22]Cardiac MRI only if abnormal cardiac ultrasound; [23]Blood tests: haemoglobin, white blood cell count, lymphocyte count, neutrophil count, platelets, sodium, potassium, creatinine, glucose, haemoglobin A1c, total bilirubin, alanine aminotransferase, aspartate aminotransferase, gamma glutamyl transpeptidase, albumin, lactate dehydrogenase, ferritin, creatine kinase, fibrinogen, INR, partial thromboplastin time, D-dimer, NT-pro-BNP, troponin, C-reactive protein (CRP), procalcitonin, venous lactate. [24]Urine tests: pH, concentration, protein, glucose, red blood, white blood cell count. [25]CD4 lymphocyte count; HIV-viral load; AIDS status. [26]HIV-therapy: drug name and dose (only ongoing treatment); previous switch to other regimens for virological failure. [27]General nursing home; residential home; specialised LTCFs; mixed LTCFs, other LTCFs; overall number of beds; ownership of the facility: public,

for profit, not for profit. [28]Questionnaires to address cognitive status: Cognitive Failure Questionnaire (CFQ), Mini-Mental State Examination, Clinical Dementia rating Scale. [29]Results of SARS-CoV-2 molecular test on amniotic fluid. [30]Weight, height/length, cranial circumference, BMI. [31]Type of transplant (hearth, lung, kidney, liver, pancreas); single-combined; year of transplantation. [32]Graft function: good, impaired, failure, rejection acute-chronic, recurrence of underlying disease, other. [33]Immunosuppressive regimen: drug name and dose. [34]According to Common Terminology Criteria for Adverse Events (CTCAE).

SECTION S3: ADDITIONAL RESULTS

Table S4. Univariable analysis of biochemical parameters associated with the occurrence of PCC divided per time point (only significant associations are shown).

	Acute infection			Month 6			Month 12			Month 18		
**PCC**	**yes**	**no**	***p-value***	**yes**	**no**	***p-value***	**yes**	**no**	***p-value***	**yes**	**no**	***p-value***
CRP (mg/L)	86.7	58.3	<0.001				3.7	2.4	<0.001	3.7	2.7	<0.001
PCT (ng/mL)										0.1	0.0	0.020
LDH (U/L)										208.7	197.9	<0.001
Albumin (g/L)				44.8	44.1	0.010						
Ferritin (ng/mL)				113.1	138.4	<0.001	122.7	140.1	<0.001			
AST (U/L)	46.3	38.8	0.020				22.7	24.0	<0.001			
ALT (U/L)										27.1	24.9	0.010
GGT (U/L)				22.4	24.5	0.050	22.8	25.6	0.010			
Total bilirubin (mg/dL)							0.5	0.6	<0.001			
Glucose (mg/dL)				112.8	104.1	0.030						
A1c Haemoglobin (mmol/mol)				37.3	39.5	<0.001						
Creatinine (mg/dL)				0.8	0.9	<0.001	0.8	0.9	<0.001			
Sodium (meq/L)	136.7	138.4	<0.001									

D-dimer (ng/mL [FEU])				338.3	474.1	<0.001	322.6	421.7	<0.001	327.2	493.7	<0.001
NT-pro-BNP (pg/mL)										76.8	122.8	0.020
Haemoglobin (g/L)				135.7	138.7	<0.001						
Leukocytes (10^9/L)							6.5	6.2	0.020	6.6	6.2	<0.001
Neutrophils (10^9/L)										4.0	3.6	<0.001
Lymphocytes (10^9/L)				1.9	1.8	<0.001	1.9	1.8	0.040	1.9	1.8	0.020
Thrombocytes (10^9/L)				245.6	235.6	<0.001	243.2	236.8	0.030	241.5	232.3	0.030

Table S5. Univariable analysis of biochemical parameters associated with the occurrence of respiratory cluster of PCC divided per time point (only significant associations are shown).

	**Acute infection**			**Month 6**			**Month 12**			**Month 18**		
**Respiratory cluster**	**yes**	**no**	***p-value***	**yes**	**no**	***p-value***	**yes**	**no**	***p-value***	**yes**	**no**	***p-value***
CRP (mg/L)	89.4	64.2	<0.001	3.6	2.7	<0.001	4.3	2.6	<0.001	4.4	2.8	<0.001
PCT ng/mL)							0.0	0.0	<0.001	0.1	0.1	0.010
LDH (U/L)	321.1	280.4	<0.001	204.6	193.5	<0.001	198.5	193.2	0.020	210.8	201.1	<0.001
Albumin (g/L)	37.7	39.4	<0.001									
Ferritin (ng/mL)	626.8	522.6	0.010									
AST (U/L)	46.8	39.9	<0.001							25.3	23.7	0.020

ALT (U/L)	40.6	33.5	<0.001	26.5	24.6	0.020						
Glucose (mg/dL)	120.7	109.5	<0.001							108.4	101.2	0.040
A1c Haemoglobin (mmol/mol)	134.7	131.8	0.010									
Creatinine (mg/dL)							0.9	0.9	0.040			
Sodium (meq/L)	136.7	137.2	0.020									
Fibrinogen (g/L)				3.4	3.1	0.040	3.4	3.2	0.020			
NT-pro-BNP (pg/mL)										63.0	111.1	<0.001
Leukocytes (10^9/L)	6.8	6.4	0.020	6.6	6.5	0.030	6.6	6.3	<0.001	6.7	6.3	0.020
Neutrophils (10^9/L)	5.1	4.7	<0.001							4.1	3.7	<0.001
Lymphocytes (10^9/L)	1.1	1.2	<0.001	2.0	1.9	0.030						
Thrombocytes (10^9/L)	217.3	205.3	0.030							248.3	232.9	<0.001

Table S6. Univariable analysis of biochemical parameters associated with the occurrence of chronic fatigue cluster of PCC divided per time point (only significant associations are shown).

	**Acute infection**			**Month 6**			**Month 12**			**Month 18**		
**Chronic fatigue cluster**	**yes**	**no**	***p-value***	**yes**	**no**	***p-value***	**yes**	**no**	***p-value***	**yes**	**no**	***p-value***
CRP (mg/L)	85.6	74.8	<0.001	3.0	2.5	0.020	3.3	2.7	<0.001	3.6	3.1	0.020

PCT ng/mL)	0.2	0.1	0.030							0.1	0.1	0.010
CK (U/L)	116.0	92.1	0.010									
Albumin (g/L)	38.7	36.3	<0.001	45.0	44.3	0.020						
Ferritin (ng/mL)				106.2	135.0	<0.001	122.7	136.9	<0.001			
AST (U/L)				21.5	22.7	0.010	22.7	23.7	<0.001			
ALT (U/L)	40.4	37.3	0.040							27.8	25.3	0.010
GGT (U/L)	46.5	52.6	0.010	21.5	24.3	0.030						
Total bilirubin (mg/dL)	0.6	0.6	<0.001				0.5	0.6	0.010			
Creatinine (mg/dL)				0.8	0.9	<0.001	0.8	0.9	<0.001			
Sodium (meq/L)	136.7	137.6	<0.001	140.4	140.0	0.050						
aPPT (RATIO)										18.8	9.0	0.040
D-dimer (ng/mL [FEU])	591.8	737.2	<0.001	351.4	423.3	0.020	311.2	401.6	<0.001	311.0	476.2	<0.001
Haemoglobin (g/L)	134.7	137.7	<0.001	134.8	138.5	<0.001	137.3	139.6	<0.001			
Leukocytes (10^9/L)										6.6	6.2	0.010
Neutrophils (10^9/L)										4.0	3.6	<0.001
Lymphocytes (10^9/L)	1.1	1.1	0.040									
Thrombocytes (10^9/L)				248.3	237.5	<0.001	245.2	237.7	0.020			

Table S7. Univariable analysis of biochemical parameters associated with the occurrence of chronic pain cluster of PCC divided per time point (only significant associations are shown).

	Acute infection			Month 6			Month 12			Month 18		
**Chronic pain cluster**	**yes**	**no**	***p-value***	**yes**	**no**	***p-value***	**yes**	**no**	***p-value***	**yes**	**no**	***p-value***
CRP (mg/L)				3.3	2.7	0.010	4.2	2.8	<0.001	3.6	3.2	0.040
PCT ng/mL)	0.2	0.2	0.010				0.0	0.0	0.010	0.1	0.1	<0.001
LDH (U/L)	284.9	316.5	<0.001							214.4	200.4	<0.001
Albumin (g/L)	40.8	37.4	<0.001									
Ferritin (ng/mL)	460.2	609.5	<0.001	81.3	134.8	<0.001	123.1	132.1	0.020	118.2	142.7	0.040
AST (U/L)				21.1	22.7	0.020	22.3	23.5	0.020			
GGT (U/L)	38.7	49.8	<0.001									
Total bilirubin (mg/dL)	0.5	0.6	<0.001									
A1c Haemoglobin (mmol/mol)	136.3	134.1	0.010									
Creatinine (mg/dL)	0.8	0.9	<0.001	0.8	0.9	<0.001	0.8	0.9	<0.001	0.9	0.9	0.020
PT (INR)	1.1	1.1	0.010									
Fibrinogen (g/L)				3.4	3.1	0.030	3.4	3.2	0.010			
D-dimer (ng/mL [FEU])	497.8	707.6	<0.001									
Haemoglobin (g/L)				134.9	137.6	0.030	136.5	139.3	<0.001			
Leukocytes (10^9/L)	6.4	6.8	<0.001									

Neutrophils (10^9/L)	4.8	5.1	0.010									
Lymphocytes (10^9/L)	1.1	1.1	0.030							1.9	1.8	0.040
Thrombocytes (10^9/L)	207.6	219.5	0.030	249.0	238.7	0.020						

Table S8. Univariable analysis of biochemical parameters associated with the occurrence of neurosensorial cluster of PCC divided per time point (only significant associations are shown).

	**Acute infection**			**Month 6**			**Month 12**			**Month 18**		
**Neurosensorial cluster**	**yes**	**no**	***p-value***	**yes**	**no**	***p-value***	**yes**	**no**	***p-value***	**yes**	**no**	***p-value***
CK (U/L)				101.9	119.0	0.020						
LDH (U/L)	285.0	314.8	<0.001	187.4	197.9	0.020						
Albumin (g/L)	40.6	36.7	<0.001									
Ferritin (ng/mL)	451.5	634.6	<0.001	87.5	130.0	<0.001	96.2	134.1	<0.001			
ALT (U/L)				21.8	25.3	<0.001						
GGT (U/L)	42.2	52.6	0.020	18.5	23.9	<0.001	19.5	25.0	<0.001	20.3	29.5	0.030
Total bilirubin (mg/dL)	0.5	0.6	<0.001				0.5	0.5	<0.001			
Glucose (mg/dL)										87.9	105.2	<0.001
A1c Haemoglobin (mmol/mol)				36.6	38.8	0.020	36.5	37.9	0.020			
Creatinine (mg/dL)	0.8	0.9	<0.001	0.8	0.9	<0.001	0.8	0.9	<0.001			
PT (INR)				1.0	1.0	0.020						

D-dimer (ng/mL [FEU])	489.0	716.6	<0.001	256.3	395.4	<0.001						
NT-pro-BNP (pg/mL)										178.1	86.5	0.040
Haemoglobin (g/L)	136.4	133.7	<0.001				134.4	139.2	<0.001			
Neutrophils (10^9/L)	4.8	5.1	0.040									
Lymphocytes (10^9/L)	1.2	1.1	<0.001									
Thrombocytes (10^9/L)	224.3	214.0	0.020									

Table S9. Univariable analysis of biochemical parameters associated with the occurrence of severe PCC divided per time point (only significant associations are shown).

	**acute**			**m6**			**m12**			**m18**		
**Severe PCC**	**yes**	**no**	***p-value***	**yes**	**no**	***p-value***	**yes**	**no**	***p-value***	**yes**	**no**	***p-value***
CRP (mg/L)				3.6	2.6	<0.001	4.4	2.9	<0.001	3.8	3.2	0.030
PCT ng/mL)				0.1	0.0	<0.001	0.1	0.0	<0.001	0.1	0.1	<0.001
LDH (U/L)	274.2	311.1	<0.001	208.0	193.2	0.010	207.5	193.5	<0.001	218.5	202.6	<0.001
Albumin (g/L)	41.0	37.5	<0.001									
Ferritin (ng/mL)	435.8	601.3	<0.001	79.3	128.3	<0.001						
ALT (U/L)				27.8	24.6	0.030						
GGT (U/L)	38.2	49.0	<0.001									
Total bilirubin (mg/dL)	0.5	0.6	<0.001				0.5	0.5	0.030			

Creatinine (mg/dL)	0.8	0.9	<0.001				0.8	0.9	0.010			
aPPT (RATIO)										22.1	11.2	0.010
PT (INR)	1.1	1.1	0.010									
Fibrinogen (g/L)							3.6	3.2	<0.001			
D-dimer (ng/mL [FEU])	487.2	701.1	<0.001									
Haemoglobin (g/L)							135.7	139.1	<0.001			
Leukocytes (10^9/L)	6.4	6.8	0.020				6.7	6.3	0.040			

Table S10. Epidemiological and demographic characteristics of panel subsamples selected for the multivariable analysis of the impact of biochemical parameters measured during the acute infection on the development of PCC and its clinical clusters.

	PCC	RESc	NSc	CPc	CFc
**Demographic**	**N (%)**	**N (%)**	**N (%)**	**N (%)**	**N (%)**
Total number of patients	176	174	180	169	176
Age	58.84 ± 12.48	58.86 ± 12.5	58.73 ± 12.4	58.84 ± 12.61	58.84 ± 12.48
Female	79 (44.9%)	77 (44.3%)	81 (45%)	75 (44.4%)	79 (44.9%)
**Underlying medical conditions**	**N (%)**	**N (%)**	**N (%)**	**N (%)**	**N (%)**
Smoker	7 (7%)	6 (6.1%)	7 (6.8%)	7 (7.4%)	7 (7%)
Pregnancy	1 (1.3%)	1 (1.4%)	1 (1.3%)	1 (1.4%)	1 (1.3%)
Diabetes	1 (0.6%)	1 (0.6%)	1 (0.6%)	1 (0.6%)	1 (0.6%)

HIV	0 (0%)	0 (0%)	0 (0%)	0 (0%)	0 (0%)
Transplant Recipients	1 (0.6%)	1 (0.6%)	1 (0.6%)	1 (0.6%)	1 (0.6%)
Auto-inflammatory Disease[1]	7 (4%)	7 (4%)	7 (3.9%)	7 (4.1%)	7 (4%)
Cardiovascular Disease[2]	65 (36.9%)	64 (36.8%)	67 (37.2%)	60 (35.5%)	65 (36.9%)
Chronic Liver Disease[3]	5 (2.9%)	5 (2.9%)	5 (2.8%)	5 (3%)	5 (2.9%)
Chronic Kidney Disease[4]	4 (2.3%)	4 (2.3%)	4 (2.2%)	4 (2.4%)	4 (2.3%)
Obesity	20 (11.4%)	20 (11.5%)	20 (11.1%)	17 (10.1%)	20 (11.4%)
Chronic Respiratory Disease[5]	22 (12.6%)	22 (12.6%)	23 (12.9%)	22 (13.2%)	22 (12.6%)
Neurological Disorders	4 (2.3%)	4 (2.3%)	4 (2.2%)	4 (2.4%)	4 (2.3%)
**Acute infection**	**N (%)**	**N (%)**	**N (%)**	**N (%)**	**N (%)**
Vaccination before acute infection	34 (24.6%)	33 (24.3%)	34 (24.1%)	33 (25.2%)	34 (24.6%)
1st Wave	25 (14.2%)	25 (14.4%)	25 (13.9%)	24 (14.2%)	25 (14.2%)
2nd Wave	9 (5.1%)	8 (4.6%)	10 (5.6%)	9 (5.3%)	9 (5.1%)
3rd Wave	96 (54.5%)	95 (54.6%)	99 (55%)	90 (53.3%)	96 (54.5%)
4th Wave	41 (23.3%)	41 (23.6 %)	41 (22.8%)	41 (24.3%)	41 (23.3%)
Hospital admission	172 (97.7%)	170 (97.7%)	176 (97.8%)	165 (97.6%)	172 (97.7%)
Intensive care unit transfer	32 (18.2%)	31 (17.8%)	34 (18.9%)	31 (18.3%)	32 (18.2%)
Overall oxygen therapy	148 (84.1%)	147 (84.5%)	152 (84.4%)	142 (84%)	148 (84.1%)
Corticosteroids administration	152 (86.4%)	151 (86.8%)	155 (86.6%)	146 (86.4%)	152 (86.4%)

PCC: post-COVID condition, RES: respiratory cluster, NSc: neurosensorial cluster, CPc: chronic pain cluster, CFc: chronic fatigue cluster; [1]rheumatic disease, inflammatory bowel disease (ulcerative colitis, Crohn's disease), psoriatic rheumatism, autoimmune hepatitis, psoriasis, atopic dermatitis, chronic urticarial, multiple sclerosis, inflammatory myopathy, systemic lupus erythematosus, systemic scleroderma, Sjögren's syndrome, Behcet's syndrome, atrophic polychondritis, antiphospholipid syndrome, Takayasu arteritis, Horton's disease, knotty periarthritis, Kawasaki's disease, microscopic polyangiitis, Wegener's disease, Churg–Strauss syndrome, rheumatoid purpura, Buerger's disease, cryoglobulinemia, sarcoidosis; [2]hypertension, congestive heart failure, coronary heart disease; [3]chronic liver disease other than cancer, including alcohol-induced liver disease, non-alcoholic fatty liver disease, non-alcoholic steatohepatitis, autoimmune hepatitis, primary biliary cirrhosis, hereditary hemochromatosis, Wilson's disease; [4]kidney damage for > 3 months; [5]asthma, chronic obstructive pulmonary disease, obstructive sleep apnoea syndrome, pulmonary hypertension, restrictive lung disease.

Table S11. Epidemiological and demographic characteristics of panel subsamples selected for the multivariable analysis of the impact of biochemical parameters measured during follow-up on the development of PCC and its clinical clusters.

	**PCC**	**RESc**	**NSc**	**CPc**	**CFc**
**Demographic**	**N (%)**	**N (%)**	**N (%)**	**N (%)**	**N (%)**
Total number of patients	947	940	1008	973	890
Age	55.2 ± 14.3	55.2 ± 14.3	55.3 ± 14.3	55.1 ± 14.3	55.1 ± 14.3
Female	504 (53.2%)	501 (53.3%)	526 (52.2%)	519 (53.3%)	476 (53.5%)
**Underlying medical conditions**	**N (%)**	**N (%)**	**N (%)**	**N (%)**	**N (%)**
Smoker	78 (8.9%)	78 (8.9%)	83 (8.8%)	81 (8.9%)	74 (9%)
Pregnancy	3 (0.6%)	3 (0.6%)	5 (1%)	5 (1%)	3 (0.7%)
Diabetes	2 (0.2%)	2 (0.2%)	2 (0.2%)	2 (0.2%)	2 (0.2%)
HIV	9 (1%)	9 (1%)	10 (1%)	10 (1%)	9 (1%)
Transplant recipients	10 (1.1%)	11 (1.2%)	11 (1.1%)	11 (1.1%)	9 (1%)
Auto-Inflammatory Disease[1]	55 (5.8%)	54 (5.7%)	58 (5.8%)	58 (6%)	53 (6%)

Cardiovascular Disease[2]	379 (40.2%)	376 (40.1%)	406 (40.3%)	387 (40%)	354 (40%)
Chronic Liver Disease[3]	27 (2.9%)	27 (2.9%)	28 (2.8%)	28 (2.9%)	25 (2.8%)
Chronic Kidney Disease[4]	26 (2.8%)	27 (2.9%)	28 (2.8%)	27 (2.8%)	24 (2.8%)
Obesity	60 (6.3%)	60 (6.4%)	65 (6.4%)	64 (6.6%)	58 (6.5%)
Chronic Respiratory Disease[5]	137 (14.5%)	137 (14.6%)	148 (14.7%)	145 (15%)	126 (14.2%)
Neurological Disorders	40 (4.2%)	40 (4.3%)	39 (3.9%)	40 (4.1%)	39 (4.4%)
**Acute infection**	**N (%)**	**N (%)**	**N (%)**	**N (%)**	**N (%)**
Vaccination before acute infection	166 (23.5%)	166 (23.6%)	170 (22.6%)	167 (22.9%)	161 (24.7%)
1st Wave	178 (18.8%)	175 (18.5%)	198 (19.6%)	183 (18.8%)	141 (15.8%)
2nd Wave	223 (23.5%)	221 (23.5%)	234 (23.2%)	229 (23.5%)	217 (24.4%)
3rd Wave	280 (29.6%)	280 (29.8%)	299 (29.7%)	291 (29.9%)	270 (30.3%)
4th Wave	157 (16.6%)	155 (16.5 %)	162 (16.1%)	158 (16.2%)	154 (17.3%)
Hospital admission	389 (41.1%)	388 (41.3%)	427 (42.4%)	408 (41.9%)	341 (38.3%)
Intensive care unit transfer	91 (9.6%)	90 (9.6%)	102 (10.1%)	91 (9.4%)	80 (9%)
Overall oxygen therapy	325 (34.3%)	323 (34.4%)	349 (35.2%)	339 (35%)	285 (32.2%)
Corticosteroids administration	379 (40.2%)	375 (39.9%)	397 (40.4%)	394 (40.5%)	359 (40.3%)

PCC: post-COVID condition, RES: respiratory cluster, NSc: neurosensorial cluster, CPc: chronic pain cluster, CFc: chronic fatigue cluster; [1]rheumatic disease, inflammatory bowel disease (ulcerative colitis, Crohn's disease), psoriatic rheumatism, autoimmune hepatitis, psoriasis, atopic dermatitis, chronic urticarial, multiple sclerosis, inflammatory myopathy, systemic lupus erythematosus, systemic scleroderma, Sjögren's syndrome, Behcet's syndrome, atrophic polychondritis, antiphospholipid syndrome, Takayasu arteritis, Horton's disease, knotty periarthritis, Kawasaki's disease, microscopic polyangiitis, Wegener's disease, Churg–Strauss syndrome, rheumatoid purpura, Buerger's disease, cryoglobulinemia, sarcoidosis; [2]hypertension, congestive heart failure, coronary heart disease; [3]chronic liver disease other than cancer, including alcohol-induced liver disease, non-alcoholic fatty liver disease, non-alcoholic steatohepatitis, autoimmune hepatitis, primary biliary cirrhosis, hereditary hemochromatosis, Wilsons' disease; [4]kidney damage for > 3 months; [5]asthma, chronic obstructive pulmonary disease, obstructive sleep apnoea syndrome, pulmonary hypertension, restrictive lung disease.

Table S12. Multivariable model including biochemical parameters measured during the acute infection, correlated with the occurrence of PCC and the distinct clusters.

	PCC (N=176)				RESc (N=174)				CFc (N=178)				CPc (N=175)				NSc (N=179)			
	OR	LB	HB	*p*	OR	LB	HB	*p*	OR	LB	HB	*p*	OR	LB	HB	*p*	OR	LB	HB	*p*
INSERM																				
UNIBO					0.61	0.28	1.31	0.20												
Age 41-60																	8.21	1.46	46.20	0.02
Age 31-40									7.88	0.84	73.98	0.07								
Female sex	3.58	1.86	6.9	< 0.001					2.84	1.46	5.54	< 0.01								
Obesity	4.16	1.25	13.89	< 0.05	2.87	1.03	7.99	< 0.05												
Former smoker																	2.54	0.62	10.04	0.2
Second wave																	7.67	1.1	53.59	< 0.05
Monoclonal antibodies*																	14.57	3.0	70.81	<0.001
Embolic events													5.36	1.44	19.89	0.01				
Other events					2.65	0.74	9.41	0.13												
General symptoms					0.15	0.01	0.74	0.13									0.06	0.002	1.27	0.07
Chronic respiratory disease					2.15	0.81	5.69	0.13												
Total bilirubin									0.37	0.23	0.59	0.16								

*PCC: post-COVID condition, RES: respiratory cluster, NSc: neurosensorial cluster, CPc: chronic pain cluster, CFc: chronic fatigue cluster; *administration during the acute infection*
